# Positive childhood experiences and subjective well-being among university students in Yunnan Province, China: The mediating role of psychological resilience

**DOI:** 10.1371/journal.pone.0352258

**Published:** 2026-06-24

**Authors:** Yanni Huang, Fuhua Yang, Keli Yin

**Affiliations:** 1 Yunnan Vocational College of Transportation, Kunming, China; 2 Faculty of Education, Yunnan Normal University, Kunming, China; 3 Yunnan University of Chinese Medicine, Kunming, China; University of the Witwatersrand, SOUTH AFRICA

## Abstract

This study examines the impact of Positive Childhood Experiences (PCEs) on the Subjective Well-Being (SWB) of university students in Yunnan Province, China, with psychological resilience as a mediating factor. Data were collected from 1,104 students across four universities using the Positive Childhood Experiences Scale, the Connor-Davidson Resilience Scale (CD-RISC), and measures of SWB. Descriptive statistics and mediation analysis were conducted using SPSS and the PROCESS macro. The results indicate that PCEs have a significant positive effect on SWB, and this relationship is partially mediated by psychological resilience. The indirect effect of PCEs on SWB through resilience was statistically significant. These findings highlight the importance of fostering resilience to enhance well-being in educational contexts. Given the regional focus on Yunnan Province, caution is warranted when generalizing the findings. Future research should examine broader and more diverse populations.

## 1. Introduction

**Subjective Well-Being (SWB)** is a key indicator of psychological health, reflecting individuals’ overall evaluation of life satisfaction and emotional states [1]. In recent years, increasing attention has been paid to SWB due to rising mental health challenges among university students worldwide, including stress, anxiety, and depression [2]. Higher levels of SWB are associated with better academic performance, healthier social relationships, and more positive career development [3]. However, surveys indicate that college students—particularly those in China—exhibit lower levels of SWB, facing challenges such as academic pressure, employment stress, and societal expectations, which have increasingly contributed to psychological health issues [4,5].

These challenges are not unique to China; similar trends have been observed globally. Therefore, it is important to examine factors that influence SWB among university students. Positive Childhood Experiences (PCEs), such as emotional support and a sense of safety during childhood, are known to contribute to long-term emotional well-being [6,7]. Furthermore, psychological resilience, which helps individuals effectively cope with challenges, plays an essential role in maintaining and enhancing SWB [8].

**Positive Childhood Experiences (PCEs)**, sometimes referred to as Benevolent Childhood Experiences (BCEs), refer to beneficial experiences occurring before the age of 18, such as positive relationships with parents and other adults, family routines, and a sense of security within the home environment [6,7]. Studies have shown that PCEs contribute to the development of emotion regulation, coping strategies, and psychological resilience—all essential for maintaining well-being later in life [9]. Converging evidence suggests that supportive early relationships are associated with healthier prefrontal–limbic functioning and lower stress reactivity, which are in turn linked to higher well-being in adulthood [10]. Longitudinal studies likewise indicate that higher-quality parent–child interactions predict meaningful gains in life satisfaction over time [11,12].

According to theories in Developmental Psychology and Positive Psychology, emotional support and a sense of security obtained during childhood form the foundation for development of trust and autonomy, which are crucial for mental health and SWB in adulthood [13,14]. Furthermore, Bowlby [13] emphasizes that secure relationships in childhood foster emotional stability and psychological resilience, which are essential for later well-being. Positive Psychology also highlights that early positive experiences, such as supportive family interactions, provide the psychological resources needed for growth and flourishing [14]. In addition, Developmental Psychopathology Theory underscores that adaptive and maladaptive pathways established in childhood critically shape resilience and well-being trajectories throughout life [15,16]. Together, these theoretical frameworks provide a comprehensive understanding of how PCEs contribute to SWB.

PCEs not only enhance SWB directly, but may also influence well-being indirectly through other psychological mechanisms [7]. For instance, positive sibling relationships, particularly the warmth dimension, contribute to emotional regulation efficacy (*β* = 0.41), which enhances resilience and, in turn, improves adult SWB [17]. PCEs are particularly relevant for college students, who face multiple stressors, such as academic pressures, transitions to independent living, and social integration challenges. According to resilience theory, PCEs foster the development of psychological resources—such as emotional regulation, coping competence, and resilience—that help individuals manage stress and recover from adversity [6,18,19]. Through these mechanisms, resilience serves as a psychological buffer that translates the benefits of early positive experiences into sustained well-being and mental health [20,21].

**Psychological Resilience** refers to an individual’s ability to recover quickly and maintain mental health when faced with stress and adversity [22]. Resilience is a crucial component of mental health, as high levels of resilience enable individuals to better adapt to various life challenges, maintaining psychological stability and well-being [23]. Several factors influence psychological resilience, including genetic factors, personal traits, social support, and environmental influences [18,22]. Research has shown that social support and positive environmental influences significantly enhance resilience. For example, Masten [18] highlighted that supportive relationships and a stable environment are key factors in fostering resilience. Additionally, studies by Fletcher and Sarkar [22] emphasize that personal traits, such as optimism and self-esteem, contribute to higher levels of resilience and well-being in the face of adversity. Theoretically, the environment and events an individual experiences during development play a crucial role in shaping their resilience. Bronfenbrenner posits that family and social environments play a crucial role in fostering an individual’s psychological resilience [24].

Recent research has increasingly emphasized that PCEs not only buffer the detrimental impact of Adverse Childhood Experiences (ACEs) but also function as independent promotive factors in developing psychological resilience. A systematic review and meta-analysis have confirmed that PCEs are significantly and positively associated with resilience in adulthood (*β* = 0.42, *p* < 0.001), and this relationship remains robust even after adjusting for ACE exposure [11,25]. Key protective dimensions of PCEs include emotional support within the family (*OR* = 2.31), high-quality peer relationships (*OR* = 1.89), and a strong sense of community belonging (*OR* = 1.65), all of which contribute meaningfully to resilience development [26]. Meta-analytical findings also suggest that the promotive effect of PCEs (e.g., enhancing mental health and social adjustment) occurs more frequently than their compensatory role in mitigating the effects of adversity [25]. Empirical studies further substantiate the significant impact of PCEs on psychological resilience, supporting the idea that early positive experiences contribute to the development of resilience and overall well-being.

Resilient individuals tend to exhibit greater emotional regulation, stress tolerance, and interpersonal competence, which together contribute to higher well-being and lower psychopathological risk [27]. For example, Chen [8] found that psychological resilience has a direct and positive effect on SWB among Chinese college students. Similarly, other studies have confirmed that PCEs can boost resilience, which subsequently enhances well-being outcomes in emerging adults [20].

Empirical evidence supports the mediating role of resilience in the PCEs–SWB link. For example, Kocatürk and Çiçek [20] examined the relationship between PCEs and psychological resilience in university students. Their findings indicated that PCEs significantly enhance psychological resilience, suggesting that individuals with PCEs are more likely to demonstrate greater resilience in the face of adversity. Similarly, Sibel et al. [21] explored how psychological flexibility and meaning-based coping mediate the relationship between PCEs and spiritual well-being, further supporting the idea that psychological resilience plays a crucial role in well-being outcomes. A study reported that psychological resilience mediated the relationship between PCEs and life satisfaction with a sizable indirect effect of 0.58 [28], demonstrating the practical relevance of this mediation model.

Although numerous studies have examined subjective well-being among Chinese university students, most have been conducted in eastern and coastal regions, while empirical evidence from southwestern provinces remains limited [29–31]. Given the substantial socio-economic and educational differences across regions in China, it remains unclear whether these relationships can be generalized to less-studied areas such as Yunnan Province. To address this gap, the present study examines these relationships among university students from four institutions in Yunnan Province, China. By focusing on a geographically underrepresented region, this study provides regionally grounded evidence on the PCE–resilience–SWB pathway.

In addition, this study contributes to the literature in two important ways. First, it extends existing research by testing the mediating role of psychological resilience in a southwestern Chinese context, thereby improving the external validity of previous findings. Second, by incorporating key socio-demographic variables—place of origin, year of study, and family income—into the mediation model, this study provides a more rigorous examination of whether the proposed mechanism remains robust after controlling for potential confounding factors.

Building on the above rationale, this study aims to examine the relationships among PCEs, psychological resilience, and SWB among university students in China. In particular, the study investigates both the direct effect of PCEs on SWB and the indirect effect through psychological resilience. Based on existing literature and relevant theoretical frameworks, the following hypotheses are proposed:

H1: PCEs positively predict SWB among college students.
H2: PCEs positively predict psychological resilience among college students.
H3: Psychological resilience positively predicts SWB among college students.
H4: Psychological resilience mediates the relationship between PCEs and SWB among college students.

**Fig 1:**
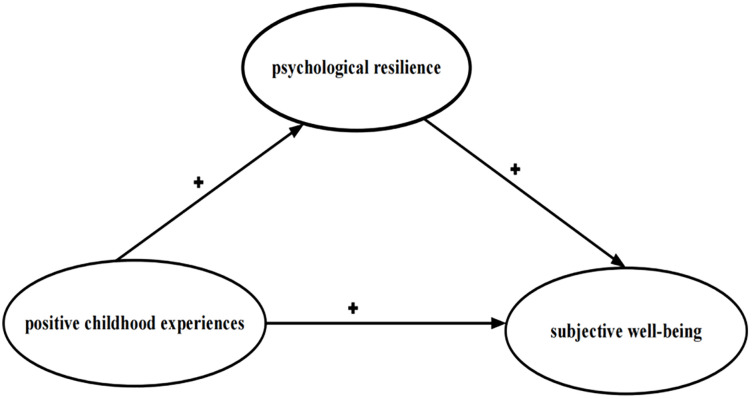
Mediation model of positive childhood experiences.

In light of the foregoing, this study presents the hypothesized model as shown in Fig 1.

## 2. Materials and methods

### 2.1. Participants and procedures

This study employed a stratified cluster random sampling method, targeting college students from four universities in Yunnan Province, China. A total of 1,220 questionnaires were distributed, and after excluding responses showing patterned or straight-line answering (e.g., selecting the same option for all items) and other invalid questionnaires, a total of 1,104 valid responses were obtained, yielding an effective response rate of 90.49%. The participants’ ages ranged from 18 to 24 years (*M* = 19.67, *SD* = 1.38). The sample included 306 male students (27.7%) and 798 female students (72.3%). In addition to age and gender, several other demographic variables were collected, including year of study, place of origin (urban/rural), only-child status, academic major, father’s and mother’s educational attainment, family structure (e.g., single-parent or nuclear family), and family income. Preliminary analyses indicated that gender, only-child status, academic major, parents’ education levels, and family structure did not show significant differences in relation to the dependent variable (subjective well-being). However, place of origin, year of study, and family income were significantly associated with SWB and were therefore included as control variables in the mediation analysis. This approach ensured that the observed mediation effects were not confounded by these demographic factors.

Data collection was conducted using the online platform “Questionnaire Star” (https://www.wjx.cn/). Participation was voluntary and anonymous, and no personally identifiable information was collected. The study protocol was reviewed by the Ethics Review Group of the Applied Psychology Program, Faculty of Education, Yunnan Normal University, and was granted an ethics exemption on August 5, 2023 (No. 2023012). The study involved a fully anonymous, non-invasive questionnaire survey of adult university students, collected no directly or indirectly identifiable personal information or sensitive personal information, and posed no greater than minimal risk to participants. Participants read the informed consent statement before voluntarily completing and submitting the anonymous questionnaire, which constituted informed consent under this study design. Participants were informed that they could withdraw at any time without providing any reason.

### 2.2. Measures

The study utilized three standardized scales to measure Positive Childhood Experiences (PCEs), psychological resilience and Subjective Well-Being (SWB). All measures were administered in Chinese, with previously validated versions of the scales.

#### 2.2.1. Positive Childhood Experiences Scale.

The Positive Childhood Experiences Scale, developed by Narayan et al. [6] consists of 10 items measuring positive experiences before the age of 18. These items are categorized into three domains: self-awareness, supportive relationships, and positive, predictable life events.

Sample items include “Did you have at least one caregiver with whom you felt safe?”, “Did you have at least one good friend?”, and “Did you like yourself or feel comfortable with yourself?”.

Respondents rate the frequency of these experiences on a binary scale (0 = “no”, 1 = “yes”). A summed score (range 0–10) was used, with higher scores indicating more PCEs. The Cronbach’s alpha for this sample was 0.77, indicating good internal consistency. All items are listed in Appendix A.

#### 2.2.2. Psychological Resilience Scale.

Psychological resilience was measured using the Connor-Davidson Resilience Scale (CD-RISC) [32]. The CD-RISC consists of items assessing resilience across several domains, including adaptability, personal competence, and the ability to cope with stress. Respondents rate each item on a 5-point Likert scale (0 = “not true at all”, 4 = “true nearly all the time”), with higher scores indicating greater resilience, as defined by the scoring guidelines of the CD-RISC. The scale has shown good psychometric properties in Chinese populations. In this study, the Cronbach’s alpha was 0.94. All items are provided in Appendix A.

#### 2.2.3 Subjective Well-Being Scale.

Subjective Well-Being was assessed using the Satisfaction with Life Scale (SWLS) [33] and the Positive and Negative Affect Schedule (PANAS) [34]. The SWLS comprises 5 items assessing global life satisfaction on a 7-point Likert scale. Positive Affect (PA) and Negative Affect (NA) were measured as affective components of well-being [35].

Following the tripartite model of SWB [36,37], which conceptualizes SWB as comprising both cognitive (life satisfaction) and affective (positive and negative affect) dimensions, we computed a composite SWB index that integrates these components.

Scores for each scale were standardized, and a composite trait SWB index was computed using the following formula: SWB = general PA – general NA + life satisfaction.

This computation method follows the empirical approach of Weinstein and Ryan [38], which demonstrated that a standardized composite index effectively captures overall trait-level well-being while maintaining balance across affective and cognitive domains.

The composite SWB score thus reflects both affective balance (PA − NA) and cognitive evaluation (life satisfaction). All components showed satisfactory internal consistency (*Cronbach’s α* = 0.88). Full items for the SWLS and PANAS are presented in Appendix A.

### 2.3. Statistical analysis

Data were analyzed using SPSS 24.0 and Hayes’ PROCESS macro (Model 4) [39] for mediation analysis. Descriptive statistics (means, SDs) were used to describe sample characteristics. Pearson correlation analyses were conducted to assess associations among the main variables. The mediating effect of psychological resilience in the relationship between PCEs and SWB was tested using 5,000 bootstrap samples. Confidence intervals were set at 95%; indirect effects were considered significant if the confidence interval (*CI)* did not include zero.

Based on preliminary analyses, year of study, place of origin, and family income were included as covariates in the mediation model to control for potential confounding variables.

## 3. Results

### 3.1. Common method bias test

Given the use of self-report measures, Harman’s single-factor test was conducted to assess potential common method bias. An exploratory factor analysis was performed on all items from the three scales. The results revealed that eight factors had eigenvalues greater than 1, and the first common factor accounted for 30.78% of the total variance, which is below the critical threshold of 40%. These results indicate that common method bias was not a serious concern in this study.

### 3.2. Descriptive results and correlation analysis

Descriptive statistics and Pearson correlation coefficients were computed to examine the relationships among PCEs, psychological resilience, and SWB (see Table 1). The correlation between PCEs and psychological resilience was 0.42 (*p* < 0.01), indicating that students with more PCEs tend to have higher levels of resilience. Similarly, the correlation between PCEs and SWB was 0.45 (*p* < 0.01), suggesting that PCEs are positively related to well-being. Moreover, the correlation between psychological resilience and SWB was 0.63 (*p* < 0.01), indicating a strong positive association between resilience and SWB.

**Table 1:** Descriptive statistics and correlations among main variables (*N* = 1104).

Variable	Mean ± SD	Min	Max	1	2	3
1. Positive Childhood Experiences	8.28 ± 2.06	0	10	1		
2. Psychological resilience	82.17 ± 15.88	32	125	0.42**	1	
3. Subjective Well-Being	0.00 ± 1.00	−4.08	3.60	0.45**	0.63**	1

*p < 0.05, **p < 0.01, ***p < 0.001. The same notation applies to the following tables.

### 3.3. Mediation analysis

To examine whether psychological resilience mediates the relationship between PCEs and SWB, a mediation analysis was conducted using Hayes’ PROCESS macro (Model 4) with 5,000 bootstrap samples and 95% confidence intervals.

**Fig 2:**
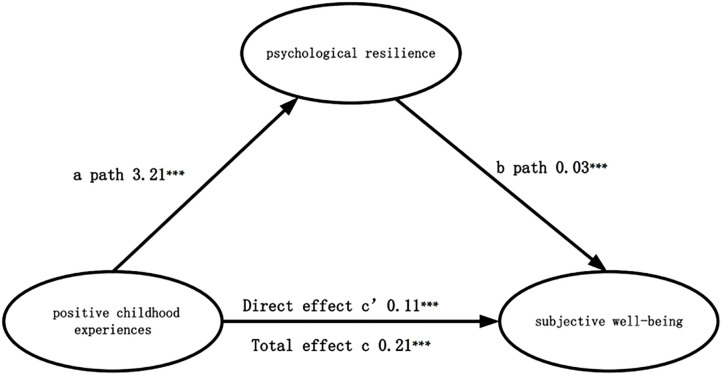
Path diagram illustrating the mediating effect of psychological resilience in the relationship between Positive Childhood Experiences and Subjective Well-Being.

Fig 2 displays the tested mediation model of PCEs, psychological resilience, and SWB. The model shows that PCEs significantly influence resilience, and resilience significantly affects SWB. For detailed numerical results, please refer to Table 2. The analysis revealed that PCEs significantly predicted SWB (*b* = 0.21, *p* < 0.001), indicating that individuals who reported more positive experiences during childhood tended to report higher levels of SWB in emerging adulthood. This total effect encompasses both the direct and indirect influences of PCEs on SWB.

**Table 2:** Path coefficients and effect sizes for each segment of the mediation model.

Path	*b*	*SE*	*t*	95% *CI*
PCEs → Resilience (a path)	3.21	0.211	15.19***	[2.79, 3.62]
Resilience → SWB (b path)	0.03	0.001	21.51***	[0.03, 0.03]
PCEs → SWB (direct effect, c’)	0.11	0.012	9.16***	[0.08, 0.13]
PCEs → SWB (total effect, c)	0.21	0.013	16.72***	—
Indirect effect (a × b)	0.10	0.009	—	[0.09, 0.13]

When psychological resilience was introduced into the model as a mediator, the direct effect of PCEs on SWB remained statistically significant (*b* = 0.11, *p* < 0.001), suggesting that PCEs exert an independent influence on SWB even after accounting for resilience.

The path from PCEs to psychological resilience was also significant (*b* = 3.21, *p* < 0.001), implying that individuals with more positive early experiences tend to exhibit greater psychological resilience. In turn, resilience significantly predicted SWB (*b* = 0.03, *p* < 0.001), showing that resilience is associated with enhanced well-being outcomes.

The indirect effect of PCEs on SWB through resilience was estimated at 0.10, with a 95% bootstrap confidence interval ranging from 0.09 to 0.13. As the confidence interval did not include zero, this indirect pathway was deemed statistically significant.

These findings indicate that psychological resilience serves as a key mediating mechanism through which PCEs contribute to enhanced SWB in young adulthood. This highlights the dual significance of early developmental environments and individual adaptive capacities in influencing long-term mental health outcomes.

## 4. Discussion

This study examined the relationships among Positive Childhood Experiences (PCEs), psychological resilience, and Subjective Well-Being (SWB) among university students in Yunnan Province, China. In addition to confirming previous findings, this study provides novel regional evidence from southwestern China, a context that has been underrepresented in well-being research. By incorporating key demographic variables—place of origin, year of study, and family income—into the mediation model, this study also provides a more rigorous test of whether the PCE–resilience–SWB pathway remains significant after controlling for socio-economic influences. The results indicated that this mediation mechanism remained robust, suggesting that the relationship between PCEs, resilience, and well-being persists even when socio-demographic factors are taken into account.

The findings are broadly consistent with resilience theory, which suggests that positive developmental environments contribute to the formation of adaptive coping systems that support psychological adjustment later in life [6,18]. In particular, the results indicate that PCEs contribute to higher levels of SWB both directly and indirectly through psychological resilience. This pattern supports the view that resilience functions as a key psychological mechanism linking early positive experiences with later well-being outcomes [20,40].

### 4.1. Experiences and college students’ subjective well-being

The results of this study indicate that PCEs are significantly associated with higher levels of SWB among college students. Students who reported more supportive and positive experiences during childhood also reported higher life satisfaction and more positive affect. This finding is consistent with previous research demonstrating that early supportive environments contribute to better psychological adjustment and higher well-being in adulthood [7,41].

However, the present study extends previous research by demonstrating that this association remains evident among university students in southwestern China. Most previous studies examining SWB among Chinese university students have focused on eastern or coastal regions, whereas empirical evidence from southwestern areas remains relatively limited. By examining students from four universities in Yunnan Province, this study provides additional regional evidence supporting the positive relationship between childhood experiences and well-being.

One possible explanation is that positive early-life experiences provide enduring psychological resources that continue to shape emotional functioning during emerging adulthood. Supportive family relationships, emotional security, and stable developmental environments may foster self-confidence, emotional stability, and positive interpersonal expectations. These developmental resources may help individuals interpret life challenges more positively and maintain higher levels of well-being.

### 4.2. The Relationship between positive childhood experiences and psychological resilience

The results of this study show that PCEs are significantly associated with psychological resilience among college students. This finding is consistent with previous research highlighting the role of positive childhood experiences in shaping an individual’s ability to cope with stress and adversity [7].

PCEs often involve experiences such as emotional support from caregivers, stable family relationships, and positive social interactions. These experiences may foster the development of effective emotional regulation strategies, problem-solving skills, and a stronger sense of personal competence. Such psychological resources are central components of resilience, enabling individuals to recover from adversity and maintain psychological stability when facing challenges [20].

For college students, resilience is particularly important because the university period is often characterized by multiple stressors, including academic pressure, social adjustment, and uncertainty about future career paths. Students who experienced more positive childhood environments may enter university with stronger internal coping resources, allowing them to adapt more effectively to these stressors. In this sense, the present findings support the view that positive developmental environments contribute to the formation of resilience, which in turn promotes psychological well-being later in life.

### 4.3. The relationship between psychological resilience and subjective well-being

The results of the study indicate a significant positive correlation between psychological resilience and SWB among college students. Psychological resilience positively influences SWB, meaning that individuals with higher psychological resilience tend to have stronger SWB. This finding is consistent with existing literature, emphasizing the critical role of psychological resilience in enhancing emotional well-being and life satisfaction [42].

Psychological resilience provides strategies for emotional regulation and problem-solving, enabling individuals to maintain a stable emotional state when facing stress and adversity. This ability helps to reduce the impact of negative emotions, enhance the experience of positive emotions, and thereby improve SWB. As a result, resilience not only protects individuals from psychological distress but also actively promotes well-being and life satisfaction [23].

Previous studies conducted among Chinese university students have reported similar findings. For example, research has shown that resilience significantly predicts SWB among college populations and plays an important role in promoting psychological adjustment during the university years [8,43]. These findings reinforce the importance of resilience as a key psychological resource that supports both mental health and SWB.

### 4.4. The mediating role of psychological resilience

The results of this study provide robust evidence that psychological resilience partially mediates the relationship between PCEs and SWB. The mediation analysis showed that PCEs significantly predicted psychological resilience, which in turn significantly predicted SWB. At the same time, the direct effect of PCEs on SWB remained significant after resilience was included in the model. This pattern indicates a partial mediation effect, suggesting that resilience explains an important part of the association between PCEs and well-being, but does not fully account for the relationship.

This finding provides further insight into the mechanisms linking early positive experiences and well-being in young adulthood. PCEs may contribute to the development of resilience by providing emotional security, supportive relationships, and opportunities to develop adaptive coping skills [44,45]. These psychological resources enable individuals to respond more effectively to stress and maintain psychological stability, which in turn contributes to higher levels of well-being.

At the same time, the persistence of a significant direct effect suggests that additional mechanisms may also link PCEs and SWB. For example, PCEs may foster stronger attachment relationships, greater perceived social support, or more positive self-concepts, all of which may independently contribute to higher well-being later in life [46]. Future research could further examine these potential pathways to develop a more comprehensive understanding of how early experiences shape psychological well-being.

From a practical perspective, these findings indicate the importance of both early developmental environments and resilience-building interventions. While positive childhood environments provide an important foundation for later well-being, resilience can also be strengthened through educational and psychological interventions during adolescence and early adulthood. Universities may therefore consider implementing programs such as resilience training, peer-support initiatives, and mindfulness-based interventions to help students better cope with academic and social stressors.

These findings have important implications for promoting mental health and well-being among college students. The results suggest that fostering positive childhood environments can have long-lasting benefits for both emotional and psychological health, not only enhancing well-being during childhood but also impacting mental health and life satisfaction in adulthood. As such, educators and family members should prioritize emotional support and positive interactions during early childhood, creating a safe and supportive environment for growth.

For college students, cultivating psychological resilience is essential for managing the stressors and challenges they face, particularly academic and social pressures. This study suggests that psychological resilience plays a key role in promoting SWB. Therefore, educational institutions should focus on developing resilience-building strategies such as mindfulness training, peer support programs, and resilience workshops. These targeted interventions can help students strengthen their emotional regulation and coping skills, enabling them to better navigate the pressures of college life.

Furthermore, fostering a supportive environment in both family and educational settings is crucial. Schools and universities should collaborate with families to ensure that students not only receive academic guidance but also the emotional support necessary to develop psychological resilience. By nurturing resilience through both early childhood experiences and ongoing interventions, institutions can help students build a strong foundation for long-term well-being and success.

## 5. Limitations and future research directions

Although this study explored the mediating role of psychological resilience between PCEs and SWB, there are still some limitations.

**Retrospective nature of the data:** The data on PCEs were collected through retrospective self-reports, which may be subject to recall bias. While retrospective self-reporting is a commonly used method, future studies could employ multiple data sources, such as interviews, parental reports, or diary methods, to cross-validate participants’ recollections and provide a more accurate assessment of childhood experiences. Additionally, experimental methods or longitudinal designs could help further validate the causal connections between PCEs and SWB.

**Cross-sectional data:** This study utilized cross-sectional data, which limits the ability to draw conclusions about causal relationships between variables. Future research could adopt a longitudinal design to track changes in psychological resilience and well-being over time. A longitudinal study would allow for a clearer understanding of how PCEs influence psychological resilience and, subsequently, SWB at different developmental stages. This approach would strengthen the ability to infer causal relationships and explore the long-term effects of childhood experiences.

**Geographic and demographic limitations:** The study sample was limited to college students from four universities in Yunnan Province, China, which may limit the generalizability of the findings to other populations. Given the regional specificity of the sample, caution should be exercised when generalizing these results to all Chinese university students. Future research could broaden the sample scope by including students from various regions across China, as well as participants from different cultural, socioeconomic, and demographic backgrounds, to enhance the external validity and generalizability of the results. Additionally, the sample was considered as a whole without examining how individual differences, such as age and gender, might moderate the studied relationships. Given the predominance of female participants in the sample, future research should explicitly explore whether gender differences influence the associations among PCEs, psychological resilience, and SWB. Moreover, age-related differences, stemming from varied life challenges and coping mechanisms, might also moderate these relationships, thus necessitating future studies to consider age-specific analyses. Therefore, a broader geographic sample should be considered to assess the generalizability of the results to other regions in China.

**Focusing on specific aspects of childhood experiences:** This study broadly examined PCEs, but future studies could explore more specific aspects of PCEs within family and school environments. Research could focus on how specific parenting styles, quality of peer relationships, or school support systems foster resilience. Identifying these specific aspects would provide useful information for developing more targeted interventions aimed at enhancing psychological resilience and improving well-being.

## 6. Conclusion

This study empirically examined the relationships between PCEs, psychological resilience, and SWB, with a specific focus on the mediating role of psychological resilience. The results indicated that PCEs significantly enhance college students’ SWB both directly and indirectly by increasing psychological resilience. This highlights the importance of fostering PCEs as a means of promoting emotional well-being during adulthood.

Furthermore, psychological resilience was found to play a key mediating role in the relationship between PCEs and SWB, emphasizing that resilience serves as a mechanism that enhances well-being by enabling individuals to cope with adversity. These findings support the understanding that psychological resilience is not only a buffer against negative life experiences but also an active contributor to maintaining and improving mental health and SWB in adulthood, particularly during the college years when students face increased social and academic pressures.

These findings provide evidence for the importance of PCEs as foundational to psychological resilience, which in turn facilitates higher SWB. Educational institutions and families should prioritize fostering resilience in children through emotional support, secure environments, and positive social relationships to improve long-term outcomes for mental health.
