# Supplementary material for: Positive childhood experiences and subjective well-being among university students in Yunnan Province, China: The mediating role of psychological resilience
Source: PLoS One. 2026 Jun 24;21(6):e0352258. doi: 10.1371/journal.pone.0352258 (PMC13293391; doi:10.1371/journal.pone.0352258)
Supplement: Attachment — Submitted filename: Response to Reviewers.docx [file pone.0352258.s002.docx]

**Response to Reviewers and Editorial Office**

**Response to Journal Requirements:**

We sincerely thank the Editorial Office for the detailed guidance regarding manuscript preparation and compliance with PLOS ONE’s publication standards. Below, we respond point-by-point to the additional journal requirements provided:

**• 1.Requirement:** Please ensure that your manuscript meets PLOS ONE's style requirements, including those for file naming.

**Response:**
We have thoroughly revised the manuscript to conform to the formatting standards outlined in the PLOS ONE templates. The main body of the manuscript and the title/author/affiliation sections have been reformatted accordingly. All uploaded files have been renamed to comply with PLOS ONE's file naming conventions.

**• 2.** **Requirement:** In the ethics statement in the Methods, you have specified that verbal consent was obtained. Please provide additional details regarding how this consent was documented and witnessed, and state whether this was approved by the IRB.

**Response:**
We appreciate the opportunity to clarify this point. In the revised “Participants and Procedures” section, we have elaborated on the informed consent procedure. Prior to participation, all students received both written and verbal explanations of the study’s aims, procedures, and their rights, including the right to withdraw at any time. Verbal informed consent was obtained in the presence of class counselors, who acted as impartial witnesses. This consent procedure was reviewed by the Ethics Committee of Yunnan Normal University and received formal exemption due to the non-invasive, anonymous, and observational nature of the study. The exemption approval code is: 2023012. We have ensured that the revised manuscript clearly reflects this information.

**•** 3. **Requirement:** Please remove all personal information and re-upload a fully anonymized data set.

**Response:**

We fully understand the importance of data privacy and transparency. The dataset has been carefully reviewed, and all columns containing any potentially identifiable or sensitive information have been completely removed—not merely hidden. The revised dataset is fully anonymized and aligns with the participants’ consent and PLOS ONE’s Data Availability and Human Participant Data policies. We have also double-checked to ensure that no hidden metadata remains.

****Reviewer #1 Comments and Responses****

We sincerely thank Reviewer #1 for their constructive and insightful feedback. We have carefully revised the manuscript in response to each of the comments provided. Detailed responses are as follows:

**• Comment 1**:

*"The introduction should highlight that the well-being of university students is a worldwide concern, as evidenced by global trends in mental health challenges within this population. Including references to studies or data that reflect these international patterns could strengthen the rationale for exploring the constructs and relationships examined in this study."*

**Response**:

We thank the reviewer for this important suggestion. In response, we revised the Introduction to emphasize that the mental health and well-being of university students is not only a national concern in China but also a pressing global issue. We incorporated relevant international data and research findings to reflect the worldwide prevalence of mental health challenges among university students. These additions position the study within a broader global context and reinforce the significance of investigating positive childhood experiences (PCEs), psychological resilience, and subjective well-being (SWB) in this population.

**• Comment 2:**

*"Improve the clarity and logical flow of the introduction by separating the theoretical background from the study’s hypotheses and objectives. A clearer structure could involve presenting the background on each key construct considered in the study—such as PCEs, psychological resilience, and SWB—in distinct paragraphs, followed by a dedicated paragraph titled The Present Study, where the authors explicitly outline the study’s objectives, hypotheses, and the mediation model."*

**Response**:

We appreciate the reviewer’s guidance. The Introduction has been thoroughly restructured to improve clarity and logical coherence. Specifically, we now present the theoretical background for each key construct—PCEs, psychological resilience, and SWB—in clearly separated paragraphs. Following these sections, we added a dedicated paragraph, which outlines the study’s theoretical framework, specific research objectives, proposed mediation model, and four hypotheses (H1–H4). This revised structure provides a coherent transition from theory to empirical investigation, better guiding the reader through the study's rationale.

**• Comment 3**:

*"Clarify why these experiences are particularly relevant to the college student population. Elaborate on how PCEs may buffer common stressors faced by students—such as academic pressure, life transitions, or new social environments."*
**Response**:

Thank you for this helpful suggestion. In response, we elaborated in the Introduction on why positive childhood experiences (PCEs) are especially relevant to college students. We now include a detailed explanation of how PCEs, by enhancing coping resources and psychological resilience, may act as protective factors against common stressors in university life, such as academic pressure, such as academic pressure, life transitions, and social adaptation. These revisions strengthen the rationale for focusing on this specific population and clarify the unique importance of PCEs in supporting their well-being.

**• Comment 4**:

“*In the Methods section, I recommend reporting the internal consistency (e.g., Cronbach’s α) for the Positive Childhood Experiences Scale, as has been done for the other instruments.*”

**Response**:

We appreciate the reviewer’s attention to detail. We have now added the Cronbach’s α coefficient for the Positive Childhood Experiences Scale in the Methods section. This ensures consistency in the psychometric reporting across all scales used in the study.

**• Comment 5**:

*“The title of the section 'Mediation Analysis of Psychological Resilience' could be misleading... I suggest simplifying the title to 'Mediation Analysis' to avoid potential confusion.”*

**Response**:

We agree with this suggestion and have revised the section title to ‘Mediation Analysis’ to more accurately reflect the content and avoid any ambiguity.

**• Comment 6**:

*“Restructure the mediation paragraph to reduce redundancy, clarify whether coefficients refer to total or direct effects, and improve readability.”*

**Response**:

We carefully revised the mediation paragraph to clarify the interpretation of each coefficient, explicitly distinguishing between total, direct, and indirect effects. Redundant phrasing has been eliminated to streamline the presentation and enhance readability.

**• Comment 7**:

*“Clarify the role of Table 2 in the mediation analysis and improve the structure of the results presentation.”*

**Response**:

We thank the reviewer for this observation. We revised the Results section to clearly explain the role of Table 2, indicating that it presents key regression coefficients from each path in the mediation model. We also reorganized the presentation to follow a logical order—total effect, effect on the mediator, and then direct/indirect effects—consistent with standard mediation analysis reporting.

**• Comment 8**:

*“Consider consolidating mediation results into a single table or follow PROCESS macro style for clarity.”*

**Response**:

Following the reviewer’s suggestion, we consolidated the mediation analysis results into a single summary table using a format inspired by the PROCESS macro output. This table clearly displays all relevant coefficients, confidence intervals, and significance levels in a concise layout.

**• Comment 9**:

*“The concluding sentence of the mediation section should come after the full presentation of the results.”*

**Response**:

We agree with the reviewer. The concluding sentence—summarizing the partial mediation effect—has been repositioned to appear after the complete results presentation, improving the logical flow of this section.

**• Comment 10**:

*“Expand the discussion of findings in the context of college students, particularly how resilience and PCEs may buffer stressors like academic and social pressures.”*

**Response**:

The Discussion section has been expanded to specifically address the relevance of the findings to the college student population. We elaborated on how resilience, fostered by PCEs, may help mitigate common stressors such as academic demands, adjustment to independence, and social transition challenges.

**• Comment 11**:

*“Provide more concrete suggestions for educational interventions that promote resilience and well-being.”*

**Response**:

We appreciate this helpful recommendation. In response, we have included several practical recommendations for university-based mental health interventions, including peer support groups, resilience-building workshops, and policies aimed at fostering supportive learning environments.

**• Comment 12**:

*“Include a brief summary tying together the findings from H1–H4.”*

**Response**:

At the end of the Discussion section, we now provide a concise summary of findings from hypotheses H1 through H4, illustrating how they collectively support the proposed mediation model and advance the study’s objectives.

**• Comment 13**:

*“I recommend expanding the limitations section to address the influence of individual differences and demographic characteristics on the study’s findings.”*

**Response**:

We thank the reviewer for this valuable suggestion. In response, we have revised the limitations section to acknowledge the potential moderating effects of age and gender. We also emphasize the need for future research to explore subgroup differences to improve the interpretation and generalizability of the findings.

****Reviewer #2 Comments and Responses****

**We sincerely thank Reviewer #2 for their thorough and constructive feedback. We have carefully revised the manuscript in accordance with each suggestion to improve the theoretical depth, logical structure, and presentation quality. Our point-by-point responses are provided below:**

**• Comment 1：**

***“****In order to be able to achieve the scholarly level of a published manuscript in PLOS ONE, the framework and logic of the manuscript should be improved.****”***

**Response**:

**We sincerely appreciate this important observation. In the revised manuscript, we have substantially restructured the Introduction section to clarify the theoretical foundations and ensure a more coherent logical progression. The research hypotheses are now clearly derived from the literature review and theoretical models, resulting in a more rigorous and scholarly framework.**

**• Comment 2：**

***“****Updating and expanding the literature references to include more studies would situate the study****”***

**Response**:

Thank you for the suggestion. We have expanded the literature review to include recent and relevant empirical studies, meta-analyses, and cross-cultural research (e.g., Olga et al., 2024; Radhika et al, 2024; Han et al., 2023;Zhang, 2025), providing a more robust scholarly context for our research.

**• Comment 3 & 4：**

***“****To enhance readability and accessibility, a few adjustments to presentation and structure could also be considered.****”***

**Response**:

We fully agree with this suggestion. The manuscript has been revised for greater clarity and coherence. Specifically, we improved paragraph transitions, refined the organization of each section to enhance readability.

**• Comment5：**

***“Formatting and details of manuscripts need to be more standardized.”***

**Response**:

Thank you for this observation. We have carefully reviewed and revised the entire manuscript to ensure consistent formatting of citations, tables, figures, and headings, in accordance with PLOS ONE’s formatting guidelines.

**• Comment 6：**

***“Line 38 The manuscript lacks a clear theory to support the relationship between the research variables.”***

**Response**:

We thank the reviewer for this observation. We have now incorporated Bronfenbrenner’s ecological systems theory and Seligman’s positive psychology framework to support the hypothesized relationships between Positive Childhood Experiences, psychological resilience, and subjective well-being.

**• Comment 7：**

***“Line 38 and line 49, the manuscript contains a large number of statements that are not supported by references.”***

**Response**:

We thank the reviewer for highlighting this issue. We have carefully reviewed the manuscript and added relevant citations to support all key statements, particularly those regarding the theoretical and empirical foundations of PCEs, resilience, and well-being.

**• Comment 8：**

***“*** *The logic of the research hypothesis is presented in a way that is difficult to understand.****”***

**Response**:

Thank you for pointing this out. We have rewritten the hypotheses section to clarify the logical flow from literature to hypothesis development. Each hypothesis (H1–H4) is now explicitly stated and logically connected to the preceding literature review, improving both clarity and coherence.

**• Comment 9&10：**

***“*** *Line 102-140, in terms of the overall structure of the manuscript, this paragraph doesn't quite fit/ is unnecessary.****”***

**Response**:

Thank you for the feedback. The paragraph in question has been removed. Content that was previously misaligned with the main narrative has been either deleted or merged to improve structural consistency.

**• Comment 11：**

***“Line 114, I am of the opinion that an ethical statement about the research would be inappropriate.”***

**Response**:

**Thank you for this perspective. As per PLOS ONE’s ethical guidelines, an ethics statement is required. We have revised the statement to be concise and factual, noting that the study was anonymous and non-invasive, and that ethical approval was formally waived by the Ethics Committee of Yunnan Normal University (approval code: 2023012).**

**• Comment 12：**

***“The materials and methods section, academic level and rigour of writing needs to be strengthened.”***

**Response**:

We fully agree. In the revised manuscript, we have significantly strengthened the Materials and Methods section by providing more detailed descriptions of the sampling procedure, measurement tools, reliability coefficients (e.g., Cronbach’s α), and statistical analyses (e.g., bootstrap method, normality testing).

**• Comment 13：**

***“Line 188, this sentence is incomprehensible.”***

**Response**:

Thank you for identifying this issue. We have rephrased the problematic sentence in the Results section to clearly convey the statistical findings, including beta coefficients, standard errors, and confidence intervals.

**• Comment 14：**

***“ In the full text, I didn't find Table 3.”***

**Response**:

We apologize for this oversight. We have corrected the table numbering throughout the manuscript. The current version now includes properly labeled Table 1 (Descriptive Statistics), Table 2 (Mediation Model Results), and Figure 2 (Mediation Path Diagram).

**• Comment 15：**

***“The Results section is too simple and needs to be made more precise in how it's presented.”***

**Response**:

The Results section has been significantly revised to include detailed statistical reporting, including indirect effects, effect sizes, bootstrapped confidence intervals, and visual model diagrams, providing a more precise and robust interpretation of the findings.

**• Comment 16：**

***“The logic of the discussion section is unclear, and I think the introduction and discussion are two different sections.”***

**Response**:

We appreciate this important feedback. We have expanded the Results section to include comprehensive statistical information, such as indirect and total effects, effect sizes, bootstrapped confidence intervals, and graphical illustrations of the mediation model.

**• Comment 17：**

***“ Line 286, I am concerned that the assumptions and data analysis of the current study do not support this conclusion.”***

**Response**:

We appreciate this critical observation. In the Conclusion and Discussion, we now emphasize that our results demonstrate associative relationships, not causality, and explicitly acknowledge the limitations of cross-sectional data.

**• Comment 18：**

***“ Line 298, I don't think the conclusions of the current study can be described as ' influence'.”***

**Response**:

Thank you for this observation. We have revised our language to replace 'influence' with more appropriate terms such as 'association' or 'relationship' throughout the manuscript, to accurately reflect the non-causal nature of our study design.

**• Comment 19：**

***“ Line 357, I think it's unappropriate for the current study to draw this conclusion.”***

**Response**:

We thank the reviewer for this careful critique. The final conclusions have been reworded to be more tentative and cautious. We now explicitly state that, while the results support our theoretical model, further longitudinal and experimental research is needed to confirm the observed relationships.
