# Supplementary material for: Positive childhood experiences and subjective well-being among university students in Yunnan Province, China: The mediating role of psychological resilience
Source: PLoS One. 2026 Jun 24;21(6):e0352258. doi: 10.1371/journal.pone.0352258 (PMC13293391; doi:10.1371/journal.pone.0352258)
Supplement: Attachment — Submitted filename: Response_to_Reviewers_auresp_2.docx [file pone.0352258.s003.docx]

We sincerely thank both reviewers for their valuable comments and constructive suggestions, which have greatly helped us improve the quality and clarity of the manuscript. Below, we provide detailed point-by-point responses to each of the reviewers’ comments. Revisions made in the manuscript are marked using blue font for easy reference.

Reviewer #1 Comments and Responses

Thank you very much for your thoughtful and constructive feedback. We appreciate your recognition of the improvements made in the manuscript and are grateful for the additional suggestions you provided. We have carefully addressed each of your points, and below are our responses to the specific comments you raised:

·Comment 1:

-Supporting Reference for Buffering Effect of PCEs

In the Introduction, the statement that “PCEs may act as a buffer against these stressors by enhancing emotional regulation, problem-solving skills, and resilience” is important, but it currently lacks an appropriate reference. Please add a supporting citation or clarify the source for this assertion.

Response: Thank you for your suggestion. We have now added a supporting reference to the Introduction section to substantiate the statement regarding the buffering effect of PCEs. The reference has been included, which specifically discusses how PCEs contribute to enhancing emotional regulation, problem-solving skills, and resilience. We believe this addition improves the scientific rigor of the manuscript.

·Comment 2:

-Figure 1 Caption: Model Terminology

The caption currently reads “Hypothetical Model of Positive Childhood Experiences.” Given that the figure depicts the tested mediation model (corresponding to PROCESS Model 4), I suggest revising the caption to “Tested Model of Positive Childhood Experiences” or “Mediation Model of Positive Childhood Experiences” to more accurately reflect its role in the study.

Response:

Thank you for your valuable suggestion. We have revised the caption of Figure 1 to “Mediation Model of Positive Childhood Experiences” to more accurately reflect its role in the study, aligning with the tested mediation model (PROCESS Model 4). This revision ensures that the terminology is clear and consistent with the rest of the manuscript.

·Comment 3:

-Redundancy in Reporting Path Coefficients

The results section presents the same path coefficients both in the text and in Table 2, sometimes with very similar wording. To avoid redundancy, I recommend reporting the numerical results in Table 2 only, while briefly describing their interpretation in the main text.

Response:

We appreciate your comment regarding redundancy. In response, we have revised the Results section to present the numerical path coefficients only in Table 2. We have removed the repetition of these coefficients in the main text, which now briefly describes the interpretation of the results, with Table 2 serving as the source for the detailed numerical values. This revision helps streamline the presentation and avoids unnecessary repetition.

·Comment 4:

-Organization and Redundancy in Results Sections (Sections 3.3 and 3.4)

The coefficients and results for the mediation analysis are repeated multiple times across Section 3.3, Section 3.4, and the figure caption. The title “Visualization of the Mediation Model” (Section 3.4) is also potentially misleading, as this section essentially repeats the results already presented. I suggest integrating all numerical results and interpretations into a single section (e.g., Section 3.3 “Mediation Analysis”), with the figure used for visualization only. The main text can refer to the figure as needed, but without repeating the statistical values.

Response:

Thank you for your thoughtful suggestion. We have revised the Results section to integrate all mediation analysis results into Section 3.3 “Mediation Analysis”, as you recommended. The figure is now used solely for visualization purposes, and the main text refers to the figure without repeating the statistical values. This revision has improved the structure and flow of the manuscript, reducing redundancy and ensuring that the key results are clearly presented.

Thank you once again for your detailed and constructive feedback. Your comments have helped us improve the manuscript's clarity and quality. The revisions made in response to your suggestions have contributed to a more focused and scientifically rigorous presentation of the research. We appreciate your time and effort in reviewing the manuscript, and we hope the revised version meets your expectations.

Reviewer #2 Comments and Responses

We would like to sincerely thank you for your thoughtful and detailed review of our manuscript. We greatly appreciate the time and effort you have taken to provide us with constructive feedback. After carefully reading through your comments, we have addressed each point in detail and made the necessary revisions to improve the manuscript. Your suggestions have been extremely valuable in refining the study and enhancing its clarity and depth.Below are our responses to your specific comments:

·Comment 1:

-The title of the article is “Chinese University Students”, and the source of the subjects in the manuscript is four universities in Yunnan Province, I found no evidence that the sample source was representative of the Chinese University.

Response:

Thank you for your valuable comment. In response to your suggestion, we have made several revisions to clarify the regional limitations of our sample. The title has been updated to reflect that the study was conducted with university students in Yunnan Province, China rather than implying a national sample. Additionally, we have revised the abstract to highlight that the findings are region-specific and may not be fully generalizable to all Chinese university students.
Furthermore, in the Limitations section, we emphasize the need for caution when generalizing these results and suggest that future research should include a broader, more geographically diverse sample to assess the generalizability of the findings across other regions in China. We believe these revisions improve the transparency and scope of the study.

·Comment 2:

-Line 36, The citation and presentation of references in this way is rare and difficult to read, and is used in the manuscript for references to empirical studies. Moreover, comparing collectivist cultures and individualist cultures, does natural contact refer to a culture, and what is the relationship between natural contact and the former two?

Response:

Thank you for your insightful comment. We have updated the citation style to ensure that references are presented in a way that aligns with SSCI publication standards.We have removed the cultural comparisons between collectivist and individualist cultures, as well as the discussion on natural contact, to better focus on the key factors influencing subjective well-being (SWB) in the context of Chinese university students. This ensures that the manuscript stays aligned with the core research question, specifically investigating how positive childhood experiences (PCEs) and psychological resilience influence SWB in university students.

·Comment3:

- Developmental Psychology Theory and Positive Psychology Theory are two very important theories, and I am curious as to how these two theories actually support the point of view of the article; Line 51 is also a very important theoretical support, but there is no literature to support it. In addition, I think there are other theories that can be referred to, such as developmental psychopathology theory (Cicchetti & Toth, 2005; Toth & Cicchetti, 2013).

I think that the support of theories to the research point of view needs to be completed and systematic arguments, not a one-sentence generalization.

Response:

Thank you for your valuable feedback. In response to your comment, we have added relevant literature references to support the claims made in Line 51, particularly regarding the importance of emotional support and a sense of security during childhood.

Additionally, we have expanded the theoretical argument by integrating developmental psychopathology theory (Cicchetti & Toth, 2005; Toth & Cicchetti, 2013). This theory emphasizes the role of both adaptive and maladaptive pathways established during childhood in shaping resilience and well-being trajectories across the lifespan. By incorporating this perspective along with developmental psychology and positive psychology, we have provided a more systematic and comprehensive theoretical foundation for understanding how positive childhood experiences (PCEs) influence subjective well-being (SWB) via resilience. We believe these revisions enhance the depth and coherence of the manuscript's theoretical support.

·Comment 4:

- Line 62 This is an analysis of why buffering stress is important for PCEs, but I have not found any psychological theory or literature to support it.

Response:

Thank you for your thoughtful comment. In response, we have now added relevant empirical studies to support the buffering effect of positive childhood experiences (PCEs). Specifically, the studies by Hall (2025) and Sousa et al. (2025) provide strong evidence that PCEs enhance emotional regulation and resilience, which in turn contribute to improved well-being. We believe these additions adequately support the buffering effect discussed in the manuscript.

·Comment 5:

- Line 67 As with the previous comment, I have not found any literature in support of this argument.

Response:

Thank you for your helpful comment. In response, we have added relevant literature to support the argument in Line 67 regarding the factors

·Comment 6:

- Line 69 This sentence contains a grammatical error.

Response:

Thank you for your comment. We have revised Line 69 of the original manuscript to correct the grammatical issue and improve clarity.

·Comment7:

- Still with the suggestion of theoretical support, I think it is not rigorous to support a research idea with just one sentence related to psychological theory.

Response:

Thank you for your comment. In response, we have expanded the theoretical support by integrating developmental psychopathology theory (Cicchetti & Toth, 2005; Toth & Cicchetti, 2013) along with developmental psychology and positive psychology. We have also added relevant literature and empirical evidence to support these theories, addressing the concern of relying on just one sentence. These revisions provide a more comprehensive and rigorous theoretical framework for our research.

·Comment 8:

- Line 86 It's incomprehensible that this sentence should appear here.

Response:

Thank you for your comment. In response, we have revised Line 86 of the original manuscript, which is now Line 102 after the revision. We have clarified and improved the clarity of the sentence by integrating additional theoretical and empirical support. The revised sentence now better highlights the impact of positive childhood experiences (PCEs) on psychological resilience and strengthens the overall argument.

·Comment 9:

- Line 88, this sentence seems redundant.

Response:

Thank you for your comment. Based on your suggestion, we have identified redundancy in Line 88 of the original manuscript. We have deleted this sentence in the revised version. Thank you for pointing this out.

·Comment 10:

- Line 96 recommends that manuscripts be carefully scrutinized for punctuation to meet publication requirements.

Is self-efficacy and emotional regulation the same concept as psychological resilience? I suspect that the cited literature does not support the research point of view.

Response:

Thank you for your comment. In response, we have carefully reviewed the manuscript for punctuation and made corrections to ensure compliance with publication requirements. Regarding your second point, we acknowledge that self-efficacy and emotional regulation are not the same as psychological resilience. While they are related constructs that may contribute to resilience, they represent distinct psychological mechanisms. To avoid conceptual overlap, we have revised the manuscript to clearly differentiate these concepts.

In addition, we have revised the cited literature to ensure that the references accurately support the research perspective. Specifically, we now focus on studies that highlight the role of psychological resilience in mediating the relationship between positive childhood experiences (PCEs) and subjective well-being (SWB). This revision strengthens the theoretical rigor of the manuscript and ensures that the cited evidence directly supports the argument.

·Comment11:

- Line 100 3 references to experimental studies, I only found the Turkish culture, the other one was not stated, so how did Line 100's concluding comments come about? As well I would like to know what is the significance of your repeated references to cross-cultural differences or stability to the ideas in your manuscript? Your study was conducted among Chinese subjects, how did cross-culturalization affect the Chinese subjects? Can the empirical studies cited here prove to be equally applicable in Chinese subjects?

Response:

Thank you for your comment. In response to your suggestion, we have removed the emphasis on cross-cultural validity and now focus more on the relevance of the mediation model in the context of Chinese subjects. This revision ensures that the argument is more relevant to our study population, without unnecessary cross-cultural comparisons. We believe this adjustment strengthens the clarity and applicability of the research.

Regarding your question about the applicability of the empirical studies cited in the context of Chinese subjects, we acknowledge that while the studies we cite primarily focus on populations from other cultural contexts, the core principles of psychological resilience and its mediating role in the PCEs-SWB link are likely to be applicable to Chinese subjects as well. However, we recognize the importance of cultural differences in shaping how PCEs and resilience are experienced and manifested, and as such, we emphasize that further research specific to the Chinese context is needed to fully confirm the applicability of these findings.
We believe that the concepts of psychological resilience and the relationship between PCEs and SWB are universal to some extent, but their expression and impact may vary based on cultural factors. Our study provides a valuable contribution to understanding how PCEs contribute to well-being in Chinese students, but further research is required to explore the cross-cultural applicability of these findings and how resilience plays out in other contexts.

·Comment 12:

- Line 102 I don't understand the point this paragraph is trying to make, why continue to make assumptions in a manuscript that have already been proven when it has already been written in the previous paragraph that the relationship between PCEs, psychological resilience, and SWB has already been proven?

Response:

Thank you for your comment. In response, we have revised the manuscript to remove redundancy and better reflect the focus of our study on the Chinese context. We have clarified that the relationship between PCEs, psychological resilience, and subjective well-being (SWB) has been established in previous research, and this study aims to explore how PCEs affect SWB directly and how psychological resilience mediates this relationship among Chinese university students. This revision strengthens the clarity of the study's objectives and emphasizes the unique contribution of our research.

·Comment 13:

- H1-H4 Please formulate your research hypothesis in more concise and scholarly language.

Response:

Thank you for your suggestion. In response, we have reformulated the research hypotheses (H1-H4) to present them in a more concise and scholarly manner. The revised hypotheses are now clearer and more aligned with academic writing standards.

·Comment 14:

- Line 124 Can't “obvious patterns (e.g., all the same answers)” in the manuscript represent an invalid questionnaire, and do all the same answers mean that the same options were chosen for all questions on the scale? Or are there consecutive questions in which consecutive questions are selected? And what are invalid questionnaires?

Response:

Thank you for your comment. In response to your question, we agree that obvious patterns in responses (e.g., all the same answers) can indicate invalid questionnaires, as they suggest a lack of thoughtful engagement or the possibility of response bias. By "all the same answers", we are referring to cases where participants choose the same option for all items on the scale, such as selecting "Strongly Agree" for every question, which raises concerns about the validity of the responses.

Additionally, in some cases, we also identify invalid questionnaires if participants consistently select the same options for consecutive questions, indicating a pattern of response that does not reflect genuine individual variation. To ensure data quality, we employed standard procedures to detect such patterns, and invalid questionnaires were excluded from the analysis. These typically involve responses that are overly uniform or inconsistent with the expected response patterns.
We have now clarified this point in the manuscript to better define and explain what we consider invalid questionnaires and how they were handled during data processing.

·Comment15:

- Line 126 I'm curious if you measured any other demographic variables, if there were differences or correlations in the other demographic variables, and what to do with them when analyzing the data.

Response:

Thank you for your insightful question regarding the demographic variables. In addition to gender and age, we collected data on the following demographic variables: year of study, place of origin (urban/rural), only-child status, academic major, father's educational attainment, mother's educational attainment, family structure (e.g., single-parent or nuclear family), and family income.

During the preliminary analysis, we examined the associations between these demographic variables and the primary outcome variable (subjective well-being). Gender, only-child status, academic major, parents’ education levels, and family structure did not show significant differences in relation to the dependent variable, and thus were not included in further statistical models.

However, place of origin, year of study, and family income showed statistically significant associations with the dependent variable. To ensure the robustness of the mediation analysis, we included these variables as control variables in the PROCESS macro (Model 4). This step helped to account for potential confounding effects.

We have clarified this approach in the revised manuscript.

·Comment 16:

- Methods:

I think you could have provided more details on how the subjects agreed to participate in the study,

"Verbal informed consent was obtained in the presence of class counselors who served as impartial witnesses." the subjects were all adult college students, so why didn't they sign an informed consent form?

Response:

Thank you for your comment. In response to your question, we used verbal informed consent as the method of obtaining consent for several reasons. First, all participants were adult college students, and it is common practice in research involving university students to use verbal consent, especially when the study poses minimal risk and does not involve sensitive or confidential information. Verbal consent was deemed appropriate for this study as it involved a non-invasive survey with no personal or potentially harmful questions.
To ensure ethical standards, the verbal consent was obtained in the presence of class counselors who acted as impartial witnesses. This procedure followed the university's ethical guidelines, and all participants were informed that they could withdraw from the study at any time without penalty.

·Comment 17:

- Line 143 I am not aware of any previous study in which the scale was validated.

Response:

Thank you for your comment. In response, we have clarified in the manuscript that the scales measuring PCEs, psychological resilience, and subjective well-being (SWB) are well-established and have been validated in previous studies. The references for these scales are provided later in the manuscript, where the scales are introduced in more detail. This ensures that the scales' validity and reliability are properly supported by the relevant literature.

·Comment 18:

- 2.2.2 Whether the Psychological Resilience Scale is scored as a total or mean score?

Response:

Thank you for your question. In response, we have clarified in the Methods section that the Connor-Davidson Resilience Scale (CD-RISC) is scored as a total score, which is the standard scoring method for this scale. The total score provides a comprehensive measure of psychological resilience, and we have explicitly stated this in the manuscript for clarity.

·Comment19:

- Line 157 Where does this conclusion come from?

Response:

Thank you for your comment. In response to your question, we have clarified that the conclusion regarding the relationship between higher scores and greater resilience is based on the scoring guidelines of the Connor-Davidson Resilience Scale (CD-RISC). We have also updated the Methods section to ensure that the Connor-Davidson Resilience Scale is properly referenced and explained, and the relevant reference has been included in the manuscript.

·Comment 20:

- Line 190 I think it is highly inappropriate to present this conclusion in descriptive statistics.

Response:

Thank you for your insightful comment. We realize that presenting this conclusion in the descriptive statistics section is indeed inappropriate. In response to your feedback, we have adjusted the manuscript by moving this conclusion to the Results section, where it is more appropriately placed. This revision ensures that the conclusions are drawn based on the proper analysis and are presented in the correct context, which improves the manuscript's clarity and structure.

·Comment 21:

- Line 215 I think the presentation here is inappropriate, you can express your findings in a paragraph, table, or mediator modeling diagram, and the coefficients for each pathway have been clearly presented in the table and mediator modeling diagram below. This part of the manuscript is too complicated and unclear, and I recommend reading the scientific presentation in the literature to meet PLOS ONE's publication standards.

Response:

Thank you for your constructive feedback. We appreciate your suggestion on improving the presentation of the findings. In response, we have revised this section to make the presentation clearer and more concise. We have moved the detailed findings into a table and the accompanying mediator modeling diagram, where the coefficients for each pathway are clearly displayed, making the results easier to interpret. Additionally, we have simplified the narrative to ensure the information is presented in a straightforward manner, in accordance with PLOS ONE’s publication standards. We believe these changes enhance the clarity and scientific rigor of the manuscript.

·Comment 22:

- Lines 247-249 The manuscript contains similar repetitive and insufficiently refined writing expressions, and it is recommended that the language be refined.

Response:

Thank you for your helpful comment. In response, we have refined the language in Lines 247-249 to eliminate redundancy and improve clarity. The revised version presents the findings in a more concise and precise manner while maintaining alignment with previous research.

·Comment23:

- The discussion section is very confusing, for example, the discussion of the findings is mixed with the value of the study, which seems unclear. The reasons that influenced the findings of the manuscript were also unclear, and I think there is a strong need to revisit the findings.

Response:

Thank you for your constructive feedback. In response to your comment, we have reorganized the Discussion section to clearly separate the presentation of the findings from the implications and value of the study. We also revisited the structure and flow of the discussion to make it more cohesive and focused.These revisions ensure that the Discussion section is clearer, more concise, and aligned with the publication standards of PLOS ONE.

·Comment 24:

- In addition to the above COMMENTS, I think there are some important comments: 1) The ideas expressed in the manuscript are very weak, and it is suggested to increase the understanding of the research topic and the analysis of the research data to enrich the depth of the study. 2) The introduction part of the manuscript mentions collectivism and individualism, as well as stability in cross-cultural research, but it does not appear in the conclusions of the study and the discussion part of the study in the context of different cultures. At the same time, I think it is very worthwhile to discuss in depth this mediation model proposed in the context of Chinese culture, and whether the mediation model is similar or different from individualism in the context of collectivism. In addition, as well as to my knowledge, Southwest China is a multi-ethnic region, does the multi-ethnic culture affect their understanding of PCEs, psychological resilience, and SWB. 3) I would like to know how you carried out the measurement of the subjects' positive childhood experiences, and whether you divided their positive childhood experiences over time, e.g., the questions on the scale represent their last three years, 6-12 years old, or their entire adolescence, and I think that there may be differences in the impact on well-being of positive childhood experiences at different time stages, and of different durations. 4) Seeing only two demographic variables in the manuscript, gender and age, I'm curious if other demographic variables were collected in your study, how other variables were handled in the data analysis, and whether the mediation model differed between genders is also worth exploring.

Response:

Thank you very much for your careful and responsible review, and for providing such constructive feedback. Your suggestions have been extremely helpful in improving the manuscript. Below are our detailed responses to the four points raised in your comment.

1）On Increasing the Depth of the Study:

We appreciate your suggestion to increase the depth of the study. In response, we have revised the Discussion section to further elaborate on the data analysis and provide a more comprehensive interpretation of the findings. Specifically, we have deepened the analysis of the mediating role of psychological resilience and explored how PCEs influence SWB through resilience. This revision enhances the theoretical grounding of the study and provides a more detailed understanding of the relationships between these variables.

2）On Cultural Considerations and Cross-Cultural Research:

However, as per Comment 2, we have already revised the manuscript to remove the comparisons between collectivist and individualist cultures, as well as the discussion on natural contact. These sections were removed to ensure the manuscript remains focused on the central theme: examining how PCEs and psychological resilience impact SWB in Chinese college students.

Regarding the multi-ethnic culture in Southwest China, we agree that it may influence how PCEs, psychological resilience, and SWB are understood and experienced. While this aspect was not directly addressed in our study, we have noted this as a potential area for future research, suggesting that further studies could explore how multi-ethnic culture affects these constructs.

3）On the Measurement of PCEs and Time Stages:

Thank you for your insightful comment regarding the measurement of PCEs and the potential differences in their impact at various stages of life. We agree that time stages (e.g., early childhood vs adolescence) may have different influences on SWB, and this is an important consideration for future research.

Regarding the current study, the Positive Childhood Experiences (PCEs) scale used is a well-established and widely recognized tool. As mentioned in the Methods section, this scale captures general positive childhood experiences but does not differentiate between specific time periods (e.g., early childhood vs adolescence). We acknowledge that future studies could examine how PCEs from different stages of childhood might influence SWB in distinct ways.

4）On Demographic Variables:

Thank you for your comment. In response to your comment, we clarified that demographic variables such as year of study, place of origin (urban vs. rural), only-child status, academic major, parental education levels, family structure, and family income were also collected in addition to age and gender. These variables were considered during data analysis. However, most demographic variables (e.g., gender, only-child status, academic major, parents' education, family structure) showed no significant differences or correlations with the main study variables (PCEs, psychological resilience, and SWB), and therefore were not included in the mediation model. In contrast, place of origin, year of study, and family income, which showed significant associations with the dependent variable, were included as control variables. We have updated the Data Analysis section accordingly to explain how these demographic variables were handled.

Once again, we would like to express our gratitude for your constructive feedback. The revisions made based on your comments have strengthened the manuscript, and we believe these improvements have contributed to a clearer, more robust presentation of our research. We appreciate your time and effort in reviewing the manuscript and hope the revised version meets your expectations.
