# Supplementary material for: Positive childhood experiences and subjective well-being among university students in Yunnan Province, China: The mediating role of psychological resilience
Source: PLoS One. 2026 Jun 24;21(6):e0352258. doi: 10.1371/journal.pone.0352258 (PMC13293391; doi:10.1371/journal.pone.0352258)
Supplement: Attachment — Submitted filename: renamed_e6b61.docx [file pone.0352258.s004.docx]

Additional Editor Comments:

1) Contribution Clarity: The manuscript repeatedly states that your findings are consistent with existing literature. Please clarify the novel theoretical or empirical contribution of your work.

Response:

We sincerely appreciate this valuable comment. We agree that the previous version did not make the unique contribution of our study sufficiently explicit. In the revised manuscript, we have clarified both the empirical and methodological contributions in the Introduction and Discussion sections.

First, we now emphasize the empirical contribution of our study by situating it within the geographical context of China’s higher education system. As noted in prior research, most studies on subjective well-being among Chinese university students have been conducted in eastern and coastal regions, while empirical evidence from southwestern provinces remains scarce. By analyzing data from four universities in Yunnan Province, our study helps to broaden the geographical coverage of well-being research in China and provides regionally grounded evidence on the PCE–resilience–SWB pathway. This addition enhances the external validity and contextual representativeness of our findings.

Second, in our mediation analysis, we included key socio-economic covariates—place of origin (urban/rural), year of study, and family income—to test whether the PCE–resilience–SWB mechanism would remain significant after controlling for demographic influences. The results demonstrated that this mediation pattern persisted when these socio-economic factors were controlled, indicating the robustness of the mechanism and improving the analytical rigor of our model.

2) SWB Measurement Justification: The decision to combine life satisfaction and affect into a single SWB measure requires strong theoretical or empirical support from the literature. If such support is lacking, we recommend treating them as two separate outcome variables in your analysis.

Response:

We sincerely appreciate the editor’s careful attention to the operationalization of Subjective Well-Being (SWB). In response, we have substantially revised the Methods section to provide a clearer theoretical and empirical justification for computing a composite SWB score.

Our operationalization is grounded in the tripartite model of SWB proposed by Diener (1984)[1] and further elaborated by Diener et al. (1999)[2], which conceptualizes SWB as comprising cognitive evaluations (life satisfaction) and affective components (positive and negative affect). This integrated perspective has become a widely accepted framework in the study of well-being.

Following this model and prior empirical research[2-4], we computed a composite SWB score consistent with the empirical method used by Weinstein and Ryan (2010)[5]. Specifically, scores for each scale were standardized, and the composite SWB index was calculated using the formula:

SWB = general PA – general NA + life satisfaction.

This standardized computation balances the contributions of affective and cognitive domains and has been shown to validly represent trait-level well-being across diverse samples (Weinstein & Ryan, 2010)[5]. We have clarified this method in Section 2.2.3 (Methods) of the revised manuscript and emphasized that the composite SWB score serves as a summary index integrating both affective balance (PA–NA) and cognitive evaluation (life satisfaction)

3) Variable Measurement Details: There is insufficient information on the measures for your key variables (Positive Childhood Experiences, Psychological Resilience, and SWB). Please list all survey questions for each measure, either in the main text or an appendix, to ensure clarity for a broad audience.

Response:

We appreciate the editor’s thoughtful suggestion. To enhance measurement transparency and reproducibility, we have now included the full item sets of all three key instruments—Positive Childhood Experiences (BCEs), Psychological Resilience (CD-RISC), and Subjective Well-Being (SWLS + PANAS)—in a newly added Appendix A.

The Methods section (2.2.1–2.2.3) now also provides concise descriptions of each scale, including response format, scoring procedures, reliability coefficients, and relevant references.

Minor Comment :

1) Summary Statistics: Add a table (e.g., Table 1) presenting summary statistics (observations, mean, standard deviation, min, max) for all variables.

Response：

We sincerely thank the editor for this helpful suggestion. In the revised manuscript, we have expanded Table 1 to include the minimum and maximum values for all main variables (Positive Childhood Experiences, Psychological Resilience, and Subjective Well-Being), in addition to the existing means and standard deviations.

2) Clarify "invalid questionnaires": In line 139, explain what constitutes "other invalid questionnaires" in a footnote.

Response：

We appreciate the editor’s careful attention to methodological clarity. To make our data screening criteria more explicit, we have added a footnote in Section 2.1 to specify what was meant by “other invalid questionnaires.”

3) Formatting Consistency: Check and correct inconsistent font sizes (e.g., lines 105-106, 233-236) in the entire document.

Response：

We are grateful to the editor for noticing these formatting inconsistencies.We have carefully reviewed and corrected all formatting inconsistencies throughout the manuscript, including font size and style.

4) Spacing Errors: Proofread the entire document to add missing spaces between words (e.g., line 78 before "Several factors").

Response：

We thank the editor for this observation. We have thoroughly proofread the entire manuscript and corrected all spacing issues, including missing spaces between words and after punctuation marks.

Reviewer #1:

Comment 1

The reviewer correctly highlights that the sentence “By enhancing emotional regulation, problem-solving skills, and resilience, PCEs can act as a buffer against these stressors, ultimately promoting better mental health and well-being [18,19]” appears central to the proposed mediation model. However, the cited references (particularly reference [18]) lack direct support for this mediation pathway. The reviewer requests a clearer theoretical articulation of how Psychological Resilience functions as the mechanism linking PCEs and SWB, both in the Introduction and Discussion.

Response:

We are very grateful to the reviewer for this insightful comment regarding the theoretical foundation of the mediation pathway linking Positive Childhood Experiences (PCEs), resilience, and Subjective Well-Being (SWB).

In response, we have carefully revised both the Introduction and Discussion sections to articulate more clearly how resilience functions as the psychological mechanism through which PCEs exert their positive effects on well-being.

Specifically, we have removed the ambiguous citation of Hall (2025) [former reference 18], as it did not offer sufficient empirical or theoretical grounding for the mediation pathway. In its place, we added and emphasized relevant literature that directly supports the mediating role of resilience, including:

“Masten (2001) and Luthar et al. (2000), who explain resilience as an adaptive process supported by early positive experiences;”

“Narayan et al. (2018) and Sousa et al. (2025), who show that PCEs promote psychological resilience across development;”

“Kocatürk & Çiçek (2023) and Melih & Oktay (2024), who provide empirical support for the mediating role of resilience between PCEs and well-being among university students.”

We have also revised the key sentence in the Introduction to more clearly reflect this developmental mechanism.
Furthermore, in the Discussion, we have avoided introducing unmeasured constructs (such as self-esteem or confidence) and instead focused on resilience as the empirically supported mediator.

These revisions strengthen the conceptual clarity and theoretical coherence of the mediation model, and we sincerely appreciate the reviewer’s valuable input, which has helped us refine the manuscript substantially.

Comment2

“It is not entirely clear what was included in the composite measure of SWB... The manuscript mentions ‘reverse-coded affect scores,’ but does not specify whether both Positive and Negative Affect were used (with the latter reversed), or whether only reversed Negative Affect was combined with SWLS…”

Response:

We appreciate the reviewer’s close reading and constructive suggestion. We have now revised the text to explicitly state that the composite SWB score was computed from three standardized components: Positive Affect (PA), Negative Affect (NA) (both assessed via PANAS), and Life Satisfaction (SWLS). The affective component was operationalized as PA minus NA, following the tripartite model of SWB (Diener, 1984; Watson et al., 1988)[1, 6], which conceptualizes cognitive and affective dimensions as distinct yet interrelated aspects of well-being.

To ensure consistency across instruments with different item numbers and scale ranges, each scale score was standardized before aggregation. The final SWB index was computed using the following formula: SWB = general PA – general NA + life satisfaction.

This computation follows the empirical approach of Weinstein and Ryan (2010)[5], who applied the same formula after standardizing individual scales, demonstrating its empirical validity and conceptual coherence. Importantly, we have clarified that the composite SWB serves as a theoretically informed composite index—an integrative measure of cognitive and affective well-being. This clarification has been incorporated in the revised Methods section (2.2.3).

1.	Diener E. Subjective well -being. Psychological Bulletin. 1984;95(3):542-75. doi: 10.1037/0033-2909.95.3.542. PubMed PMID: WOS:A1984SR64000009.

2.	Diener E, Suh EM, Lucas RE, Smith HL. Subjective well-being: Three decades of progress. Psychological Bulletin. 1999;125(2):276-302. doi: 10.1037//0033-2909.125.2.276. PubMed PMID: WOS:000078980900007.

3.	Argyle M, Crossland J. The dimensions of positive emotions. British Journal of Social Psychology. 1987;26(2):127-37. doi: 10.1111/j.2044-8309.1987.tb00773.x.

4.	Diener E, Emmons RA. The independence of positive and negative affect. Journal of personality and social psychology. 1984;47(5):1105. doi: 10.1037/0022-3514.47.5.1105.

5.	Weinstein N, Ryan RM. When helping helps: autonomous motivation for prosocial behavior and its influence on well-being for the helper and recipient. Journal of personality and social psychology. 2010;98(2):222. doi: 10.1037/a0016984.

6.	Watson D, Clark LA, Tellegen A. Development and validation of brief measures of positive and negative affect: the PANAS scales. Journal of personality and social psychology. 1988;54(6):1063. doi: 10.1037/0022-3514.54.6.1063.
