# Supplementary material for: Positive childhood experiences and subjective well-being among university students in Yunnan Province, China: The mediating role of psychological resilience
Source: PLoS One. 2026 Jun 24;21(6):e0352258. doi: 10.1371/journal.pone.0352258 (PMC13293391; doi:10.1371/journal.pone.0352258)
Supplement: Attachment — Submitted filename: Response_to_Reviewers_auresp_4.docx [file pone.0352258.s005.docx]

**Response to Reviewers**

We sincerely thank the Academic Editor and the reviewers for their valuable comments and constructive suggestions. We have carefully revised the manuscript in accordance with all comments. All revisions have been clearly marked in the tracked-changes version, and the specific modifications are detailed below.

We would like to sincerely thank Reviewer #1 for the positive evaluation of our manuscript and for confirming that all previous comments have been adequately addressed. We greatly appreciate the reviewer’s time and constructive feedback throughout the review process.

**Reviewer #3:**

**Comment 1:**

*Discussion section refers to other studies but in some cases these other studies are not explained in relation to this study findings.*

**Response:**

We sincerely thank the reviewer for this insightful and helpful comment, which has significantly improved the clarity and depth of the Discussion section. In response, we have carefully revised the Discussion to more clearly connect the cited literature with the findings of the present study.

Specifically, we reorganized each subsection of the Discussion to first restate the key findings of this study and then interpret these findings in relation to existing literature. We also strengthened the explanation of the underlying mechanisms linking Positive Childhood Experiences (PCEs), psychological resilience, and Subjective Well-Being (SWB).

In particular, we have substantially revised the section on the mediating role of psychological resilience to clarify how resilience functions as a pathway linking PCEs and SWB. We now explicitly explain the partial mediation effect observed in this study and discuss possible additional mechanisms that may account for the remaining direct effect.

These revisions have enhanced the theoretical interpretation of our findings and strengthened the overall contribution of the study. The revised content can be found in Section 4 (Discussion), particularly in subsections 4.1–4.4.

**Comment 2:**

*There are still grammatical errors and spacing issues within the document.*

**Response:**

We sincerely thank the reviewer for pointing this out. We have carefully proofread the entire manuscript and corrected grammatical errors and spacing issues throughout the text. In particular, we have revised punctuation, spacing between words and references, and minor typographical errors to improve clarity and readability.

**Comment 3:**

*The lack of ethics clearance is a concern as there are some items in the questionnaires that are invasive.*

**Response:**

We sincerely thank the reviewer for raising this important concern, which has helped us improve the clarity and transparency of the ethical statement.

In response, we have added a detailed ethical statement in the Methods section (Section 2.1 Participants and Procedures). The revised manuscript now clearly states that the study protocol was reviewed by the Ethics Committee of Yunnan Normal University and was granted exemption from full ethical review (Approval No. 2023012), as the study involved an anonymous questionnaire survey of adult participants and posed minimal risk.

We have also clarified that no personally identifiable information was collected. In addition, we explicitly state that participation was voluntary, informed consent was obtained prior to participation, and participants had the right to withdraw from the study at any time without penalty.

These revisions ensure that the ethical considerations of the study are clearly presented and fully comply with international publication standards.

We sincerely appreciate the editor’s and reviewers’ time and valuable feedback. We believe that the revisions have addressed all concerns and significantly improved the manuscript, and we hope that the revised version is now suitable for publication in PLOS ONE.
