# Supplementary material for: Positive childhood experiences and subjective well-being among university students in Yunnan Province, China: The mediating role of psychological resilience
Source: PLoS One. 2026 Jun 24;21(6):e0352258. doi: 10.1371/journal.pone.0352258 (PMC13293391; doi:10.1371/journal.pone.0352258)
Supplement: Attachment — Submitted filename: Response_to_Reviewers_auresp_5.docx [file pone.0352258.s006.docx]

We sincerely thank you for your careful evaluation of our manuscript and for your constructive comments and suggestions. We have carefully revised the manuscript accordingly. Below we provide our point-by-point response to the editorial comment.

**Response to the Academic Editor**

**Comment :**

Please confirm whether the study has received ethics clearance.
On page 7 of the manuscript, it states that ‘The study protocol was reviewed by the ethics committee of Yunnan Normal University.’ However, it remains unclear why the study qualified for exemption, given that it involved direct interaction with participants and the collection of informed consent, even with the use of anonymous questionnaires. Kindly upload the official communication from the ethics committee indicating that the protocol was reviewed and exempted and the reasons for exemption.

**Response:**

Thank you very much for this important comment. We agree that the ethics statement in the previous version of the manuscript was not sufficiently detailed.

We would like to clarify that this study was reviewed by the Ethics Review Group of the Applied Psychology Program, Faculty of Education, Yunnan Normal University, and was granted an ethics exemption on August 5, 2023 (No. 2023012).

The exemption was granted because the study involved a fully anonymous, non-invasive questionnaire survey. No directly or indirectly identifiable personal information was collected, no sensitive personal information was involved, and the study included only general adult university students rather than vulnerable populations. In addition, the study involved no physical, clinical, psychological, or medical intervention, and the overall level of risk was considered no greater than minimal. Participants read the informed consent statement before voluntarily completing and submitting the anonymous questionnaire, which constituted informed consent under this study design.

After receiving the editorial comment, we communicated with the university’s Ethics Review Group and obtained a formal certificate based on the original ethics review record. The certificate dated April 17, 2026 is an official documentary confirmation of the original review decision made on August 5, 2023.

To support this clarification, we have uploaded the following four attachments:

Original Ethical Review Application (English)
Original Ethical Review Application (Chinese)
Certificate of Ethics Review Exemption (Chinese)
Certificate of Ethics Review Exemption (English)
We have also revised the ethics statement in the manuscript accordingly to make the review status and the basis for exemption clearer.
Revised text in the manuscript:
“The study protocol was reviewed by the Ethics Review Group of the Applied Psychology Program, Faculty of Education, Yunnan Normal University, and was granted an ethics exemption on August 5, 2023 (No. 2023012). The study involved a fully anonymous, non-invasive questionnaire survey of adult university students, collected no directly or indirectly identifiable personal information or sensitive personal information, and posed no greater than minimal risk to participants. Participants read the informed consent statement before voluntarily completing and submitting the anonymous questionnaire, which constituted informed consent under this study design. Participants were informed that they could withdraw at any time without providing any reason.”
Location in revised manuscript:
Methods section, page 7, lines 162—170.

We thank the Academic Editor again for this helpful comment, which has enabled us to improve the clarity and transparency of the manuscript. We hope that the revised version is now suitable for publication in PLOS ONE.
