# Supplementary material for: Positive childhood experiences and subjective well-being among university students in Yunnan Province, China: The mediating role of psychological resilience
Source: PLoS One. 2026 Jun 24;21(6):e0352258. doi: 10.1371/journal.pone.0352258 (PMC13293391; doi:10.1371/journal.pone.0352258)
Supplement: Peer review history

# Peer review: Decision Letter 0

**DOI:** 10.1371/journal.pone.0352258.r001

**Authors:** Petri Böckerman

20 Dec 2024

PONE-D-24-48467

Positive Childhood Experiences and Subjective Well-being Among Chinese University Students: The Mediating Role of Resilience

PLOS ONE

Dear Dr. Yin,

Thank you for submitting your manuscript to PLOS ONE. After careful consideration, we feel that it has merit but does not fully meet PLOS ONE’s publication criteria as it currently stands. Therefore, we invite you to submit a revised version of the manuscript that addresses the points raised during the review process.

The revised version should address all comments. This is a major revision with no guarantee of the paper's acceptance.

Please submit your revised manuscript by Feb 03 2025 11:59PM. If you will need more time than this to complete your revisions, please reply to this message or contact the journal office at plosone@plos.org. When you're ready to submit your revision, log on to https://www.editorialmanager.com/pone/ and select the 'Submissions Needing Revision' folder to locate your manuscript file.

Please include the following items when submitting your revised manuscript:

- A rebuttal letter that responds to each point raised by the academic editor and reviewer(s). You should upload this letter as a separate file labeled 'Response to Reviewers'.
- A marked-up copy of your manuscript that highlights changes made to the original version. You should upload this as a separate file labeled 'Revised Manuscript with Track Changes'.
- An unmarked version of your revised paper without tracked changes. You should upload this as a separate file labeled 'Manuscript'.

If you would like to make changes to your financial disclosure, please include your updated statement in your cover letter. Guidelines for resubmitting your figure files are available below the reviewer comments at the end of this letter.

If applicable, we recommend that you deposit your laboratory protocols in protocols.io to enhance the reproducibility of your results. Protocols.io assigns your protocol its own identifier (DOI) so that it can be cited independently in the future. For instructions see: https://journals.plos.org/plosone/s/submission-guidelines#loc-laboratory-protocols. Additionally, PLOS ONE offers an option for publishing peer-reviewed Lab Protocol articles, which describe protocols hosted on protocols.io. Read more information on sharing protocols at https://plos.org/protocols?utm_medium=editorial-email&utm_source=authorletters&utm_campaign=protocols.

We look forward to receiving your revised manuscript.

Kind regards,

Petri Böckerman

Academic Editor

PLOS ONE

Journal Requirements:

When submitting your revision, we need you to address these additional requirements.

1. Please ensure that your manuscript meets PLOS ONE's style requirements, including those for file naming. The PLOS ONE style templates can be found at

https://journals.plos.org/plosone/s/file?id=wjVg/PLOSOne_formatting_sample_main_body.pdf and

https://journals.plos.org/plosone/s/file?id=ba62/PLOSOne_formatting_sample_title_authors_affiliations.pdf

2. In the ethics statement in the Methods, you have specified that verbal consent was obtained. Please provide additional details regarding how this consent was documented and witnessed, and state whether this was approved by the IRB

3. Please remove all personal information, ensure that the data shared are in accordance with participant consent, and re-upload a fully anonymized data set.

Note: spreadsheet columns with personal information must be removed and not hidden as all hidden columns will appear in the published file.

Additional guidance on preparing raw data for publication can be found in our Data Policy (https://journals.plos.org/plosone/s/data-availability#loc-human-research-participant-data-and-other-sensitive-data) and in the following article: http://www.bmj.com/content/340/bmj.c181.long.

Additional Editor Comments :

The revised version should address all comments. This is a major revision with no guarantee of the paper's acceptance.

[Note: HTML markup is below. Please do not edit.]

Reviewers' comments:

Reviewer's Responses to Questions

**Comments to the Author**

1. Is the manuscript technically sound, and do the data support the conclusions?

The manuscript must describe a technically sound piece of scientific research with data that supports the conclusions. Experiments must have been conducted rigorously, with appropriate controls, replication, and sample sizes. The conclusions must be drawn appropriately based on the data presented.

Reviewer #1: Partly

Reviewer #2: Partly

**********

2. Has the statistical analysis been performed appropriately and rigorously?

Reviewer #1: Yes

Reviewer #2: Yes

**********

3. Have the authors made all data underlying the findings in their manuscript fully available?

The PLOS Data policy requires authors to make all data underlying the findings described in their manuscript fully available without restriction, with rare exception (please refer to the Data Availability Statement in the manuscript PDF file). The data should be provided as part of the manuscript or its supporting information, or deposited to a public repository. For example, in addition to summary statistics, the data points behind means, medians and variance measures should be available. If there are restrictions on publicly sharing data—e.g. participant privacy or use of data from a third party—those must be specified.

Reviewer #1: Yes

Reviewer #2: Yes

**********

4. Is the manuscript presented in an intelligible fashion and written in standard English?

PLOS ONE does not copyedit accepted manuscripts, so the language in submitted articles must be clear, correct, and unambiguous. Any typographical or grammatical errors should be corrected at revision, so please note any specific errors here.

Reviewer #1: Yes

Reviewer #2: No

**********

5. Review Comments to the Author

Please use the space provided to explain your answers to the questions above. You may also include additional comments for the author, including concerns about dual publication, research ethics, or publication ethics. (Please upload your review as an attachment if it exceeds 20,000 characters)

Reviewer #1: This study addresses a highly relevant topic, as the well-being of college students is a matter of increasing concern. By exploring the role of Positive Childhood Experiences (PCEs) in relation to subjective well-being (SWB), with psychological resilience as a mediator, the authors aim to shed light on important mechanisms underlying mental health in this population.

Here below, I have provided a series of suggestions that I hope will be useful in further improving the clarity, structure, and interpretative depth of the manuscript.

--Introduction--

The introduction provides a solid overview of the study's background and highlights the relevance of Positive Childhood Experiences (PCEs) and their connection to subjective well-being (SWB).

The authors emphasize this issue particularly in the context of China. While this is certainly relevant, it would be helpful to highlight that the well-being of university students is a worldwide concern, as evidenced by global trends in mental health challenges within this population. Including references to studies or data that reflect these international patterns could strengthen the rationale for exploring the constructs and relationships examined in this study. This would position the research within a broader, global context and further emphasize the importance of addressing these issues.

Additionally, I suggest improving the clarity and logical flow of the introduction by separating the theoretical background from the study’s hypotheses and objectives. A clearer structure could involve presenting the background on each key construct considered in the study—such as PCEs, psychological resilience, and SWB—in distinct paragraphs, followed by a dedicated paragraph titled The Present Study, where the authors explicitly outline the study’s objectives, hypotheses, and the mediation model describing the relationships among the variables.

This approach would help streamline the flow of ideas, and better guide the reader from the theoretical background to the specific aims of the research.

While the introduction effectively explains the concept of PCEs and their general role in well-being, it would also be helpful to clarify why these experiences are particularly relevant to the college student population. For instance, elaborating on how PCEs may buffer common stressors faced by students—such as academic pressure, life transitions, or new social environments—would further strengthen the rationale for focusing on this specific group.

--Methods--

In the Methods section, I recommend reporting the internal consistency (e.g., Cronbach’s α) for the Positive Childhood Experiences Scale, as has been done for the other instruments. This would ensure consistency in the presentation of the psychometric properties across all measures used.

--Analysis and Results--

The title of the section 'Mediation Analysis of Psychological Resilience' could be misleading, as it seems to suggest that the analysis focuses on resilience as the outcome variable. In reality, the mediation analysis aims to examine subjective well-being as the dependent variable, with resilience acting as the mediator. I suggest simplifying the title to 'Mediation Analysis' to avoid potential confusion and more accurately reflect the focus of the model.

The paragraph describing the mediation analysis would benefit from restructuring to improve clarity and flow, as some parts are repetitive and create confusion. For example, the coefficient 3.15 is mentioned multiple times, but it is unclear whether it represents the total effect from a regression without the mediator or a separate analysis. Specifically, in the first mention, 'positive childhood experiences significantly predicted subjective well-being (β = 3.15, p < 0.001)', and later, 'The total effect of positive childhood experiences on subjective well-being was 3.15 (95% CI [2.78, 3.52])'. If this value represents the total effect, it should be stated clearly once and avoided elsewhere to reduce redundancy. Similarly, the direct effect of 1.60 is repeated: 'When psychological resilience was included as a mediator, the direct effect of positive childhood experiences on subjective well-being remained significant (β = 1.60, p < 0.001)' and again as 'while the direct effect was 1.60 (95% CI [1.25, 1.94])'. This duplication does not add new information and could be streamlined to improve readability.

In addition, Table 2 could be presented more clearly. If the regressions reported correspond to intermediate steps in the mediation analysis, this connection needs to be explicitly explained in the text. Presenting the results in a more structured order—starting with the total effect, then the relationship between the independent variable and the mediator, and finally the direct and indirect effects—would help clarify the flow of the analysis.

I also recommend consolidating the mediation results into a single, well-organized table to eliminate redundancy and provide a clear overview of the key findings, making the results easier to follow and interpret. Alternatively, if the authors prefer to present the results across two tables, it would be important to clearly specify which variables are predictors and which are outcomes. Taking inspiration from the output format of the PROCESS macro could provide a straightforward and widely recognized template for presenting mediation analyses.

Finally, the sentence 'These analysis suggest that psychological resilience partially mediates the relationship between positive childhood experiences and subjective well-being...', which serves as a conclusion to the mediation analysis, should be moved to the end of the section. Placing it after presenting the total, direct, and indirect effects would provide a more logical and coherent closing to the paragraph.

--Discussion--

The discussion appropriately addresses the study’s hypotheses (H1–H4) and interprets the results within an appropriate theoretical context. The authors effectively connect their findings to developmental and positive psychology frameworks, referencing existing literature to support their claims. However, I suggest further expanding the interpretation of the results beyond the current descriptions. While the mediating role of resilience (H4) is well-explained, the discussion should explore in greater depth how these findings apply to the college student population, particularly in relation to the challenges and stressors unique to this group, such as academic pressures, transitional life stages, and social stressors. This focus would better contextualize the relevance of the results and strengthen their practical significance.

Additionally, while the implications for educational interventions are mentioned, I recommend expanding this section to include more concrete and actionable suggestions. For example, the authors could discuss targeted interventions or programs designed to enhance resilience and subjective well-being in college students, such as resilience-building workshops or mental health support systems. Including such examples would make the practical contributions of the findings more tangible and impactful.

Lastly, while the key hypotheses are discussed individually, it might be helpful to include a brief summary that ties together the findings for H1–H4, reinforcing how they collectively contribute to the study’s broader objectives.

--Limitations--

I recommend expanding the limitations section to address the influence of individual differences and demographic characteristics on the study’s findings. The sample has been considered as a whole, but factors such as age and gender could potentially moderate the relationships between Positive Childhood Experiences, psychological resilience, and subjective well-being. For instance, younger and older students might report different levels of subjective well-being and resilience due to varying life challenges and coping mechanisms. Additionally, since the sample has a predominance of female participants, it would be important to acknowledge that gender differences may influence these relationships, as suggested in previous literature. Explicitly recognizing these sources of variability would provide a more nuanced interpretation of the study's findings and their generalizability.

Reviewer #2: This study explores the link between positive childhood experiences and subjective well-being in Chinese college students, finding that psychological resilience played a partially mediating role.

Nevertheless, there are some areas for improvement that could strengthen the study’s quality and clarity:

Comment 1:

- In order to be able to achieve the scholarly level of a published manuscript in PLOS ONE, the framework and logic of the manuscript should be improved.

Comment 2:

- Updating and expanding the literature references to include more studies would situate the study

Comment 3:

-To enhance readability and accessibility, a few adjustments to presentation and structure could also be considered.

Comment 4:

-To enhance readability and accessibility, a few adjustments to presentation and structure could also be considered.

Comment 5:

- Formatting and details of manuscripts need to be more standardized.

Comment 6:

- Line 38 The manuscript lacks a clear theory to support the relationship between the research variables.

Comment 7:

- Line 38 and line 49, the manuscript contains a large number of statements that are not supported by references.

Comment 8:

- The logic of the research hypothesis is presented in a way that is difficult to understand.

Comment 9:

- Line 102-140, in terms of the overall structure of the manuscript, this paragraph doesn't quite fit.

Comment 10:

- Line 102-140, in terms of the overall structure of the manuscript, this paragraph is unnecessary.

Comment 11:

- Line 114, I am of the opinion that an ethical statement about the research would be inappropriate.

Comment 12:

- The materials and methods section, academic level and rigour of writing needs to be strengthened.

Comment 13:

- Line 188, this sentence is incomprehensible.

Comment 14:

- In the full text, I didn't find Table 3.

Comment 15:

- The Results section is too simple and needs to be made more precise in how it's presented.

Comment 16:

- The logic of the discussion section is unclear, and I think the introduction and discussion are two different sections.

Comment 17:

- Line 286, I am concerned that the assumptions and data analysis of the current study do not support this conclusion.

Comment 18:

- Line 298, I don't think the conclusions of the current study can be described as ' influence'.

Comment 19:

- Line 357, I think it's unappropriate for the current study to draw this conclusion.

**********

6. PLOS authors have the option to publish the peer review history of their article (what does this mean?). If published, this will include your full peer review and any attached files.

If you choose “no”, your identity will remain anonymous but your review may still be made public.

**Do you want your identity to be public for this peer review?**   For information about this choice, including consent withdrawal, please see our Privacy Policy.

Reviewer #1: No

Reviewer #2: No

**********

[NOTE: If reviewer comments were submitted as an attachment file, they will be attached to this email and accessible via the submission site. Please log into your account, locate the manuscript record, and check for the action link "View Attachments". If this link does not appear, there are no attachment files.]

While revising your submission, please upload your figure files to the Preflight Analysis and Conversion Engine (PACE) digital diagnostic tool, https://pacev2.apexcovantage.com/. PACE helps ensure that figures meet PLOS requirements. To use PACE, you must first register as a user. Registration is free. Then, login and navigate to the UPLOAD tab, where you will find detailed instructions on how to use the tool. If you encounter any issues or have any questions when using PACE, please email PLOS at figures@plos.org. Please note that Supporting Information files do not need this step.

# Author comment: Author response to Decision Letter 1

**DOI:** 10.1371/journal.pone.0352258.r002

15 Jun 2025

Response to Reviewers and Editorial Office

Response to Journal Requirements:

We sincerely thank the Editorial Office for the detailed guidance regarding manuscript preparation and compliance with PLOS ONE’s publication standards. Below, we respond point-by-point to the additional journal requirements provided:

• 1.Requirement: Please ensure that your manuscript meets PLOS ONE's style requirements, including those for file naming.

Response:

We have thoroughly revised the manuscript to conform to the formatting standards outlined in the PLOS ONE templates. The main body of the manuscript and the title/author/affiliation sections have been reformatted accordingly. All uploaded files have been renamed to comply with PLOS ONE's file naming conventions.

• 2. Requirement: In the ethics statement in the Methods, you have specified that verbal consent was obtained. Please provide additional details regarding how this consent was documented and witnessed, and state whether this was approved by the IRB.

Response:

We appreciate the opportunity to clarify this point. In the revised “Participants and Procedures” section, we have elaborated on the informed consent procedure. Prior to participation, all students received both written and verbal explanations of the study’s aims, procedures, and their rights, including the right to withdraw at any time. Verbal informed consent was obtained in the presence of class counselors, who acted as impartial witnesses. This consent procedure was reviewed by the Ethics Committee of Yunnan Normal University and received formal exemption due to the non-invasive, anonymous, and observational nature of the study. The exemption approval code is: 2023012. We have ensured that the revised manuscript clearly reflects this information.

• 3. Requirement: Please remove all personal information and re-upload a fully anonymized data set.

Response:

We fully understand the importance of data privacy and transparency. The dataset has been carefully reviewed, and all columns containing any potentially identifiable or sensitive information have been completely removed—not merely hidden. The revised dataset is fully anonymized and aligns with the participants’ consent and PLOS ONE’s Data Availability and Human Participant Data policies. We have also double-checked to ensure that no hidden metadata remains.

Reviewer #1 Comments and Responses

We sincerely thank Reviewer #1 for their constructive and insightful feedback. We have carefully revised the manuscript in response to each of the comments provided. Detailed responses are as follows:

• Comment 1:

"The introduction should highlight that the well-being of university students is a worldwide concern, as evidenced by global trends in mental health challenges within this population. Including references to studies or data that reflect these international patterns could strengthen the rationale for exploring the constructs and relationships examined in this study."

Response:

We thank the reviewer for this important suggestion. In response, we revised the Introduction to emphasize that the mental health and well-being of university students is not only a national concern in China but also a pressing global issue. We incorporated relevant international data and research findings to reflect the worldwide prevalence of mental health challenges among university students. These additions position the study within a broader global context and reinforce the significance of investigating positive childhood experiences (PCEs), psychological resilience, and subjective well-being (SWB) in this population.

• Comment 2:

"Improve the clarity and logical flow of the introduction by separating the theoretical background from the study’s hypotheses and objectives. A clearer structure could involve presenting the background on each key construct considered in the study—such as PCEs, psychological resilience, and SWB—in distinct paragraphs, followed by a dedicated paragraph titled The Present Study, where the authors explicitly outline the study’s objectives, hypotheses, and the mediation model."

Response:

We appreciate the reviewer’s guidance. The Introduction has been thoroughly restructured to improve clarity and logical coherence. Specifically, we now present the theoretical background for each key construct—PCEs, psychological resilience, and SWB—in clearly separated paragraphs. Following these sections, we added a dedicated paragraph, which outlines the study’s theoretical framework, specific research objectives, proposed mediation model, and four hypotheses (H1–H4). This revised structure provides a coherent transition from theory to empirical investigation, better guiding the reader through the study's rationale.

• Comment 3:

"Clarify why these experiences are particularly relevant to the college student population. Elaborate on how PCEs may buffer common stressors faced by students—such as academic pressure, life transitions, or new social environments."

Response:

Thank you for this helpful suggestion. In response, we elaborated in the Introduction on why positive childhood experiences (PCEs) are especially relevant to college students. We now include a detailed explanation of how PCEs, by enhancing coping resources and psychological resilience, may act as protective factors against common stressors in university life, such as academic pressure, such as academic pressure, life transitions, and social adaptation. These revisions strengthen the rationale for focusing on this specific population and clarify the unique importance of PCEs in supporting their well-being.

• Comment 4:

“In the Methods section, I recommend reporting the internal consistency (e.g., Cronbach’s α) for the Positive Childhood Experiences Scale, as has been done for the other instruments.”

Response:

We appreciate the reviewer’s attention to detail. We have now added the Cronbach’s α coefficient for the Positive Childhood Experiences Scale in the Methods section. This ensures consistency in the psychometric reporting across all scales used in the study.

• Comment 5:

“The title of the section 'Mediation Analysis of Psychological Resilience' could be misleading... I suggest simplifying the title to 'Mediation Analysis' to avoid potential confusion.”

Response:

We agree with this suggestion and have revised the section title to ‘Mediation Analysis’ to more accurately reflect the content and avoid any ambiguity.

• Comment 6:

“Restructure the mediation paragraph to reduce redundancy, clarify whether coefficients refer to total or direct effects, and improve readability.”

Response:

We carefully revised the mediation paragraph to clarify the interpretation of each coefficient, explicitly distinguishing between total, direct, and indirect effects. Redundant phrasing has been eliminated to streamline the presentation and enhance readability.

• Comment 7:

“Clarify the role of Table 2 in the mediation analysis and improve the structure of the results presentation.”

Response:

We thank the reviewer for this observation. We revised the Results section to clearly explain the role of Table 2, indicating that it presents key regression coefficients from each path in the mediation model. We also reorganized the presentation to follow a logical order—total effect, effect on the mediator, and then direct/indirect effects—consistent with standard mediation analysis reporting.

• Comment 8:

“Consider consolidating mediation results into a single table or follow PROCESS macro style for clarity.”

Response:

Following the reviewer’s suggestion, we consolidated the mediation analysis results into a single summary table using a format inspired by the PROCESS macro output. This table clearly displays all relevant coefficients, confidence intervals, and significance levels in a concise layout.

• Comment 9:

“The concluding sentence of the mediation section should come after the full presentation of the results.”

Response:

We agree with the reviewer. The concluding sentence—summarizing the partial mediation effect—has been repositioned to appear after the complete results presentation, improving the logical flow of this section.

• Comment 10:

“Expand the discussion of findings in the context of college students, particularly how resilience and PCEs may buffer stressors like academic and social pressures.”

Response:

The Discussion section has been expanded to specifically address the relevance of the findings to the college student population. We elaborated on how resilience, fostered by PCEs, may help mitigate common stressors such as academic demands, adjustment to independence, and social transition challenges.

• Comment 11:

“Provide more concrete suggestions for educational interventions that promote resilience and well-being.”

Response:

We appreciate this helpful recommendation. In response, we have included several practical recommendations for university-based mental health interventions, including peer support groups, resilience-building workshops, and policies aimed at fostering supportive learning environments.

• Comment 12:

“Include a brief summary tying together the findings from H1–H4.”

Response:

At the end of the Discussion section, we now provide a concise summary of findings from hypotheses H1 through H4, illustrating how they collectively support the proposed mediation model and advance the study’s objectives.

• Comment 13:

“I recommend expanding the limitations section to address the influence of individual differences and demographic characteristics on the study’s findings.”

Response:

We thank the reviewer for this valuable suggestion. In response, we have revised the limitations section to acknowledge the potential moderating effects of age and gender. We also emphasize the need for future research to explore subgroup differences to improve the interpretation and generalizability of the findings.

Reviewer #2 Comments and Responses

We sincerely thank Reviewer #2 for their thorough and constructive feedback. We have carefully revised the manuscript in accordance with each suggestion to improve the theoretical depth, logical structure, and presentation quality. Our point-by-point responses are provided below:

• Comment 1：

“In order to be able to achieve the scholarly level of a published manuscript in PLOS ONE, the framework and logic of the manuscript should be improved.”

Response:

We sincerely appreciate this important observation. In the revised manuscript, we have substantially restructured the Introduction section to clarify the theoretical foundations and ensure a more coherent logical progression. The research hypotheses are now clearly derived from the literature review and theoretical models, resulting in a more rigorous and scholarly framework.

• Comment 2：

“Updating and expanding the literature references to include more studies would situate the study”

Response:

Thank you for the suggestion. We have expanded the literature review to include recent and relevant empirical studies, meta-analyses, and cross-cultural research (e.g., Olga et al., 2024; Radhika et al, 2024; Han et al., 2023;Zhang, 2025), providing a more robust scholarly context for our research.

• Comment 3 & 4：

“To enhance readability and accessibility, a few adjustments to presentation and structure could also be considered.”

Response:

We fully agree with this suggestion. The manuscript has been revised for greater clarity and coherence. Specifically, we improved paragraph transitions, refined the organization of each section to enhance readability.

• Comment5：

“Formatting and details of manuscripts need to be more standardized.”

Response:

Thank you for this observation. We have carefully reviewed and revised the entire manuscript to ensure consistent formatting of citations, tables, figures, and headings, in accordance with PLOS ONE’s formatting guidelines.

• Comment 6：

“Line 38 The manuscript lacks a clear theory to support the relationship between the research variables.”

Response:

We thank the reviewer for this observation. We have now incorporated Bronfenbrenner’s ecological systems theory and Seligman’s positive psychology framework to support the hypothesized relationships between Positive Childhood Experiences, psychological resilience, and subjective well-being.

• Comment 7：

“Line 38 and line 49, the manuscript contains a large number of statements that are not supported by references.”

Response:

We thank the reviewer for highlighting this issue. We have carefully reviewed the manuscript and added relevant citations to support all key statements, particularly those regarding the theoretical and empirical foundations of PCEs, resilience, and well-being.

• Comment 8：

“ The logic of the research hypothesis is presented in a way that is difficult to understand.”

Response:

Thank you for pointing this out. We have rewritten the hypotheses section to clarify the logical flow from literature to hypothesis development. Each hypothesis (H1–H4) is now explicitly stated and logically connected to the preceding literature review, improving both clarity and coherence.

• Comment 9&10：

“ Line 102-140, in terms of the overall structure of the manuscript, this paragraph doesn't quite fit/ is unnecessary.”

Response:

Thank you for the feedback. The paragraph in question has been removed. Content that was previously misaligned with the main narrative has been either deleted or merged to improve structural consistency.

• Comment 11：

“Line 114, I am of the opinion that an ethical statement about the research would be inappropriate.”

Response:

Thank you for this perspective. As per PLOS ONE’s ethical guidelines, an ethics statement is required. We have revised the statement to be concise and factual, noting that the study was anonymous and non-invasive, and that ethical approval was formally waived by the Ethics Committee of Yunnan Normal University (approval code: 2023012).

• Comment 12：

“The materials and methods section, academic level and rigour of writing needs to be strengthened.”

Response:

We fully agree. In the revised manuscript, we have significantly strengthened the Materials and Methods section by providing more detailed descriptions of the sampling procedure, measurement tools, reliability coefficients (e.g., Cronbach’s α), and statistical analyses (e.g., bootstrap method, normality testing).

• Comment 13：

“Line 188, this sentence is incomprehensible.”

Response:

Thank you for identifying this issue. We have rephrased the problematic sentence in the Results section to clearly convey the statistical findings, including beta coefficients, standard errors, and confidence intervals.

• Comment 14：

“ In the full text, I didn't find Table 3.”

Response:

We apologize for this oversight. We have corrected the table numbering throughout the manuscript. The current version now includes properly labeled Table 1 (Descriptive Statistics), Table 2 (Mediation Model Results), and Figure 2 (Mediation Path Diagram).

• Comment 15：

“The Results section is too simple and needs to be made more precise in how it's presented.”

Response:

The Results section has been significantly revised to include detailed statistical reporting, including indirect effects, effect sizes, bootstrapped confidence intervals, and visual model diagrams, providing a more precise and robust interpretation of the findings.

• Comment 16：

“The logic of the discussion section is unclear, and I think the introduction and discussion are two different sections.”

Response:

We appreciate this important feedback. We have expanded the Results section to include comprehensive statistical information, such as indirect and total effects, effect sizes, bootstrapped confidence intervals, and graphical illustrations of the mediation model.

• Comment 17：

“ Line 286, I am concerned that the assumptions and data analysis of the current study do not support this conclusion.”

Response:

We appreciate this critical observation. In the Conclusion and Discussion, we now emphasize that our results demonstrate associative relationships, not causality, and explicitly acknowledge the limitations of cross-sectional data.

• Comment 18：

“ Line 298, I don

Attachment Submitted filename: Response to Reviewers.docx

# Peer review: Decision Letter 1

**DOI:** 10.1371/journal.pone.0352258.r003

**Authors:** Ching Sin Siau

24 Jul 2025

PONE-D-24-48467R1

Positive Childhood Experiences and Subjective Well-being Among Chinese University Students: The Mediating Role of Resilience

PLOS ONE

Dear Dr. Yin,

Thank you for submitting your manuscript to PLOS ONE. After careful consideration, we feel that it has merit but does not fully meet PLOS ONE’s publication criteria as it currently stands. Therefore, we invite you to submit a revised version of the manuscript that addresses the points raised during the review process.

==============================

**In view of the reviewers' comments, a major revision is warranted. Please address especially reviewer 2's perspective that while the study is of value, there are necessary improvements needed regarding writing style and content presentation.**

==============================

Please submit your revised manuscript by Sep 07 2025 11:59PM. If you will need more time than this to complete your revisions, please reply to this message or contact the journal office at plosone@plos.org. When you're ready to submit your revision, log on to https://www.editorialmanager.com/pone/ and select the 'Submissions Needing Revision' folder to locate your manuscript file.

Please include the following items when submitting your revised manuscript:

- A rebuttal letter that responds to each point raised by the academic editor and reviewer(s). You should upload this letter as a separate file labeled 'Response to Reviewers'.
- A marked-up copy of your manuscript that highlights changes made to the original version. You should upload this as a separate file labeled 'Revised Manuscript with Track Changes'.
- An unmarked version of your revised paper without tracked changes. You should upload this as a separate file labeled 'Manuscript'.

If you would like to make changes to your financial disclosure, please include your updated statement in your cover letter. Guidelines for resubmitting your figure files are available below the reviewer comments at the end of this letter.

If applicable, we recommend that you deposit your laboratory protocols in protocols.io to enhance the reproducibility of your results. Protocols.io assigns your protocol its own identifier (DOI) so that it can be cited independently in the future. For instructions see: https://journals.plos.org/plosone/s/submission-guidelines#loc-laboratory-protocols. Additionally, PLOS ONE offers an option for publishing peer-reviewed Lab Protocol articles, which describe protocols hosted on protocols.io. Read more information on sharing protocols at https://plos.org/protocols?utm_medium=editorial-email&utm_source=authorletters&utm_campaign=protocols.

We look forward to receiving your revised manuscript.

Kind regards,

Ching Sin Siau

Academic Editor

PLOS ONE

Journal Requirements:

If the reviewer comments include a recommendation to cite specific previously published works, please review and evaluate these publications to determine whether they are relevant and should be cited. There is no requirement to cite these works unless the editor has indicated otherwise.

[Note: HTML markup is below. Please do not edit.]

Reviewers' comments:

Reviewer's Responses to Questions

**Comments to the Author**

1. If the authors have adequately addressed your comments raised in a previous round of review and you feel that this manuscript is now acceptable for publication, you may indicate that here to bypass the “Comments to the Author” section, enter your conflict of interest statement in the “Confidential to Editor” section, and submit your "Accept" recommendation.

Reviewer #1: All comments have been addressed

Reviewer #2: All comments have been addressed

**********

2. Is the manuscript technically sound, and do the data support the conclusions?

The manuscript must describe a technically sound piece of scientific research with data that supports the conclusions. Experiments must have been conducted rigorously, with appropriate controls, replication, and sample sizes. The conclusions must be drawn appropriately based on the data presented.

Reviewer #1: Yes

Reviewer #2: Yes

**********

3. Has the statistical analysis been performed appropriately and rigorously?

Reviewer #1: Yes

Reviewer #2: Yes

**********

4. Have the authors made all data underlying the findings in their manuscript fully available?

The PLOS Data policy requires authors to make all data underlying the findings described in their manuscript fully available without restriction, with rare exception (please refer to the Data Availability Statement in the manuscript PDF file). The data should be provided as part of the manuscript or its supporting information, or deposited to a public repository. For example, in addition to summary statistics, the data points behind means, medians and variance measures should be available. If there are restrictions on publicly sharing data—e.g. participant privacy or use of data from a third party—those must be specified.

Reviewer #1: Yes

Reviewer #2: Yes

**********

5. Is the manuscript presented in an intelligible fashion and written in standard English?

PLOS ONE does not copyedit accepted manuscripts, so the language in submitted articles must be clear, correct, and unambiguous. Any typographical or grammatical errors should be corrected at revision, so please note any specific errors here.

Reviewer #1: Yes

Reviewer #2: Yes

**********

6. Review Comments to the Author

Please use the space provided to explain your answers to the questions above. You may also include additional comments for the author, including concerns about dual publication, research ethics, or publication ethics. (Please upload your review as an attachment if it exceeds 20,000 characters)

Reviewer #1: I would like to thank the authors for the effort made in revising the manuscript and for the comprehensive responses to my comments. The revised version shows a clear improvement in structure, clarity, and overall quality. My suggestions have been addressed and implemented.

However, after a detailed review of the revised manuscript, I believe a few minor revisions are still needed to further enhance the scientific rigor and readability of the work. Please find my points below:

-Supporting Reference for Buffering Effect of PCEs

In the Introduction, the statement that “PCEs may act as a buffer against these stressors by enhancing emotional regulation, problem-solving skills, and resilience” is important, but it currently lacks an appropriate reference. Please add a supporting citation or clarify the source for this assertion.

-Figure 1 Caption: Model Terminology

The caption currently reads “Hypothetical Model of Positive Childhood Experiences.” Given that the figure depicts the tested mediation model (corresponding to PROCESS Model 4), I suggest revising the caption to “Tested Model of Positive Childhood Experiences” or “Mediation Model of Positive Childhood Experiences” to more accurately reflect its role in the study.

-Redundancy in Reporting Path Coefficients

The results section presents the same path coefficients both in the text and in Table 2, sometimes with very similar wording. To avoid redundancy, I recommend reporting the numerical results in Table 2 only, while briefly describing their interpretation in the main text.

-Organization and Redundancy in Results Sections (Sections 3.3 and 3.4)

The coefficients and results for the mediation analysis are repeated multiple times across Section 3.3, Section 3.4, and the figure caption. The title “Visualization of the Mediation Model” (Section 3.4) is also potentially misleading, as this section essentially repeats the results already presented. I suggest integrating all numerical results and interpretations into a single section (e.g., Section 3.3 “Mediation Analysis”), with the figure used for visualization only. The main text can refer to the figure as needed, but without repeating the statistical values.

These are minor issues and do not detract from the overall quality of the revision, which has addressed all the major concerns raised in the previous review. Once these points are addressed, I believe the manuscript will be suitable for publication.

Thank you again for your thorough work and responsiveness.

Best regards

Reviewer #2: The subject matter of the manuscript's research is valuable, but the manuscript's research is very weak, and the author's writing and presentation of the manuscript needs to be optimized, especially in terms of the logic and rigor of the research writing and ideas, which undermines the quality of the manuscript as a whole. The language of the manuscript also needs to be optimized to fit the standards of English writing. Most importantly, I think the authors may not have fully understood the reviewers' comments during the first revision.

Comment 1:

- The title of the article is “Chinese University Students”, and the source of the subjects in the manuscript is four universities in Yunnan Province, I found no evidence that the sample source was representative of the Chinese University.

Comment 2:

- Line 36, The citation and presentation of references in this way is rare and difficult to read, and is used in the manuscript for references to empirical studies. Moreover, comparing collectivist cultures and individualist cultures, does natural contact refer to a culture, and what is the relationship between natural contact and the former two?

Comment 3:

- Developmental Psychology Theory and Positive Psychology Theory are two very important theories, and I am curious as to how these two theories actually support the point of view of the article; Line 51 is also a very important theoretical support, but there is no literature to support it. In addition, I think there are other theories that can be referred to, such as developmental psychopathology theory (Cicchetti & Toth, 2005; Toth & Cicchetti, 2013).

I think that the support of theories to the research point of view needs to be completed and systematic arguments, not a one-sentence generalization.

References

Cicchetti, D., & Toth, S.L., 2005. Child maltreatment. Annual Review of Clinical Psychology, 1, 409–438. https://doi.org/10.1146/annurev.clinpsy.1.102803.144029

Toth, S. L., & Cicchetti, D. (2013). A developmental psychopathology perspective on child maltreatment. Child Maltreatment, 18(3), 135-139. https://doi.org/10.1177/1077559513500380

Comment 4:

- Line 62 This is an analysis of why buffering stress is important for PCEs, but I have not found any psychological theory or literature to support it.

Comment 5:

- Line 67 As with the previous comment, I have not found any literature in support of this argument.

Comment 6:

- Line 69 This sentence contains a grammatical error.

Comment 7:

- Still with the suggestion of theoretical support, I think it is not rigorous to support a research idea with just one sentence related to psychological theory.

Comment 8:

- Line 86 It's incomprehensible that this sentence should appear here.

Comment 9:

- Line 88, this sentence seems redundant.

Comment 10:

- Line 96 recommends that manuscripts be carefully scrutinized for punctuation to meet publication requirements.

Is self-efficacy and emotional regulation the same concept as psychological resilience? I suspect that the cited literature does not support the research point of view.

Comment 11:

- Line 100 3 references to experimental studies, I only found the Turkish culture, the other one was not stated, so how did Line 100's concluding comments come about? As well I would like to know what is the significance of your repeated references to cross-cultural differences or stability to the ideas in your manuscript? Your study was conducted among Chinese subjects, how did cross-culturalization affect the Chinese subjects? Can the empirical studies cited here prove to be equally applicable in Chinese subjects?

Comment 12:

- Line 102 I don't understand the point this paragraph is trying to make, why continue to make assumptions in a manuscript that have already been proven when it has already been written in the previous paragraph that the relationship between PCEs, psychological resilience, and SWB has already been proven?

Comment 13:

- H1-H4 Please formulate your research hypothesis in more concise and scholarly language.

Comment 14:

- Line 124 Can't “obvious patterns (e.g., all the same answers)” in the manuscript represent an invalid questionnaire, and do all the same answers mean that the same options were chosen for all questions on the scale? Or are there consecutive questions in which consecutive questions are selected? And what are invalid questionnaires?

Comment 15:

- Line 126 I'm curious if you measured any other demographic variables, if there were differences or correlations in the other demographic variables, and what to do with them when analyzing the data.

Comment 16:

- Methods:

I think you could have provided more details on how the subjects agreed to participate in the study,

"Verbal informed consent was obtained in the presence of class counselors who served as impartial witnesses." the subjects were all adult college students, so why didn't they sign an informed consent form?

Comment 17:

- Line 143 I am not aware of any previous study in which the scale was validated.

Comment 18:

- 2.2.2 Whether the Psychological Resilience Scale is scored as a total or mean score?

Comment 19:

- Line 157 Where does this conclusion come from?

Comment 20:

- Line 190 I think it is highly inappropriate to present this conclusion in descriptive statistics.

Comment 21:

- Line 215 I think the presentation here is inappropriate, you can express your findings in a paragraph, table, or mediator modeling diagram, and the coefficients for each pathway have been clearly presented in the table and mediator modeling diagram below. This part of the manuscript is too complicated and unclear, and I recommend reading the scientific presentation in the literature to meet PLOS ONE's publication standards.

Comment 22:

- Lines 247-249 The manuscript contains similar repetitive and insufficiently refined writing expressions, and it is recommended that the language be refined.

Comment 23:

- The discussion section is very confusing, for example, the discussion of the findings is mixed with the value of the study, which seems unclear. The reasons that influenced the findings of the manuscript were also unclear, and I think there is a strong need to revisit the findings.

Comment 24:

- In addition to the above COMMENTS, I think there are some important comments: 1) The ideas expressed in the manuscript are very weak, and it is suggested to increase the understanding of the research topic and the analysis of the research data to enrich the depth of the study. 2) The introduction part of the manuscript mentions collectivism and individualism, as well as stability in cross-cultural research, but it does not appear in the conclusions of the study and the discussion part of the study in the context of different cultures. At the same time, I think it is very worthwhile to discuss in depth this mediation model proposed in the context of Chinese culture, and whether the mediation model is similar or different from individualism in the context of collectivism. In addition, as well as to my knowledge, Southwest China is a multi-ethnic region, does the multi-ethnic culture affect their understanding of PCEs, psychological resilience, and SWB. 3) I would like to know how you carried out the measurement of the subjects' positive childhood experiences, and whether you divided their positive childhood experiences over time, e.g., the questions on the scale represent their last three years, 6-12 years old, or their entire adolescence, and I think that there may be differences in the impact on well-being of positive childhood experiences at different time stages, and of different durations. 4) Seeing only two demographic variables in the manuscript, gender and age, I'm curious if other demographic variables were collected in your study, how other variables were handled in the data analysis, and whether the mediation model differed between genders is also worth exploring.

**********

7. PLOS authors have the option to publish the peer review history of their article (what does this mean?). If published, this will include your full peer review and any attached files.

If you choose “no”, your identity will remain anonymous but your review may still be made public.

**Do you want your identity to be public for this peer review?**   For information about this choice, including consent withdrawal, please see our Privacy Policy.

Reviewer #1: No

Reviewer #2: No

**********

[NOTE: If reviewer comments were submitted as an attachment file, they will be attached to this email and accessible via the submission site. Please log into your account, locate the manuscript record, and check for the action link "View Attachments". If this link does not appear, there are no attachment files.]

While revising your submission, please upload your figure files to the Preflight Analysis and Conversion Engine (PACE) digital diagnostic tool, https://pacev2.apexcovantage.com/. PACE helps ensure that figures meet PLOS requirements. To use PACE, you must first register as a user. Registration is free. Then, login and navigate to the UPLOAD tab, where you will find detailed instructions on how to use the tool. If you encounter any issues or have any questions when using PACE, please email PLOS at figures@plos.org. Please note that Supporting Information files do not need this step.

# Author comment: Author response to Decision Letter 2

**DOI:** 10.1371/journal.pone.0352258.r004

2 Sep 2025

We sincerely thank both reviewers for their valuable comments and constructive suggestions, which have greatly helped us improve the quality and clarity of the manuscript. Below, we provide detailed point-by-point responses to each of the reviewers’ comments. Revisions made in the manuscript are marked using blue font for easy reference.

Reviewer #1 Comments and Responses

Thank you very much for your thoughtful and constructive feedback. We appreciate your recognition of the improvements made in the manuscript and are grateful for the additional suggestions you provided. We have carefully addressed each of your points, and below are our responses to the specific comments you raised:

·Comment 1:

-Supporting Reference for Buffering Effect of PCEs

In the Introduction, the statement that “PCEs may act as a buffer against these stressors by enhancing emotional regulation, problem-solving skills, and resilience” is important, but it currently lacks an appropriate reference. Please add a supporting citation or clarify the source for this assertion.

Response: Thank you for your suggestion. We have now added a supporting reference to the Introduction section to substantiate the statement regarding the buffering effect of PCEs. The reference has been included, which specifically discusses how PCEs contribute to enhancing emotional regulation, problem-solving skills, and resilience. We believe this addition improves the scientific rigor of the manuscript.

·Comment 2:

-Figure 1 Caption: Model Terminology

The caption currently reads “Hypothetical Model of Positive Childhood Experiences.” Given that the figure depicts the tested mediation model (corresponding to PROCESS Model 4), I suggest revising the caption to “Tested Model of Positive Childhood Experiences” or “Mediation Model of Positive Childhood Experiences” to more accurately reflect its role in the study.

Response:

Thank you for your valuable suggestion. We have revised the caption of Figure 1 to “Mediation Model of Positive Childhood Experiences” to more accurately reflect its role in the study, aligning with the tested mediation model (PROCESS Model 4). This revision ensures that the terminology is clear and consistent with the rest of the manuscript.

·Comment 3:

-Redundancy in Reporting Path Coefficients

The results section presents the same path coefficients both in the text and in Table 2, sometimes with very similar wording. To avoid redundancy, I recommend reporting the numerical results in Table 2 only, while briefly describing their interpretation in the main text.

Response:

We appreciate your comment regarding redundancy. In response, we have revised the Results section to present the numerical path coefficients only in Table 2. We have removed the repetition of these coefficients in the main text, which now briefly describes the interpretation of the results, with Table 2 serving as the source for the detailed numerical values. This revision helps streamline the presentation and avoids unnecessary repetition.

·Comment 4:

-Organization and Redundancy in Results Sections (Sections 3.3 and 3.4)

The coefficients and results for the mediation analysis are repeated multiple times across Section 3.3, Section 3.4, and the figure caption. The title “Visualization of the Mediation Model” (Section 3.4) is also potentially misleading, as this section essentially repeats the results already presented. I suggest integrating all numerical results and interpretations into a single section (e.g., Section 3.3 “Mediation Analysis”), with the figure used for visualization only. The main text can refer to the figure as needed, but without repeating the statistical values.

Response:

Thank you for your thoughtful suggestion. We have revised the Results section to integrate all mediation analysis results into Section 3.3 “Mediation Analysis”, as you recommended. The figure is now used solely for visualization purposes, and the main text refers to the figure without repeating the statistical values. This revision has improved the structure and flow of the manuscript, reducing redundancy and ensuring that the key results are clearly presented.

Thank you once again for your detailed and constructive feedback. Your comments have helped us improve the manuscript's clarity and quality. The revisions made in response to your suggestions have contributed to a more focused and scientifically rigorous presentation of the research. We appreciate your time and effort in reviewing the manuscript, and we hope the revised version meets your expectations.

Reviewer #2 Comments and Responses

We would like to sincerely thank you for your thoughtful and detailed review of our manuscript. We greatly appreciate the time and effort you have taken to provide us with constructive feedback. After carefully reading through your comments, we have addressed each point in detail and made the necessary revisions to improve the manuscript. Your suggestions have been extremely valuable in refining the study and enhancing its clarity and depth.Below are our responses to your specific comments:

·Comment 1:

-The title of the article is “Chinese University Students”, and the source of the subjects in the manuscript is four universities in Yunnan Province, I found no evidence that the sample source was representative of the Chinese University.

Response:

Thank you for your valuable comment. In response to your suggestion, we have made several revisions to clarify the regional limitations of our sample. The title has been updated to reflect that the study was conducted with university students in Yunnan Province, China rather than implying a national sample. Additionally, we have revised the abstract to highlight that the findings are region-specific and may not be fully generalizable to all Chinese university students.

Furthermore, in the Limitations section, we emphasize the need for caution when generalizing these results and suggest that future research should include a broader, more geographically diverse sample to assess the generalizability of the findings across other regions in China. We believe these revisions improve the transparency and scope of the study.

·Comment 2:

-Line 36, The citation and presentation of references in this way is rare and difficult to read, and is used in the manuscript for references to empirical studies. Moreover, comparing collectivist cultures and individualist cultures, does natural contact refer to a culture, and what is the relationship between natural contact and the former two?

Response:

Thank you for your insightful comment. We have updated the citation style to ensure that references are presented in a way that aligns with SSCI publication standards.We have removed the cultural comparisons between collectivist and individualist cultures, as well as the discussion on natural contact, to better focus on the key factors influencing subjective well-being (SWB) in the context of Chinese university students. This ensures that the manuscript stays aligned with the core research question, specifically investigating how positive childhood experiences (PCEs) and psychological resilience influence SWB in university students.

·Comment3:

- Developmental Psychology Theory and Positive Psychology Theory are two very important theories, and I am curious as to how these two theories actually support the point of view of the article; Line 51 is also a very important theoretical support, but there is no literature to support it. In addition, I think there are other theories that can be referred to, such as developmental psychopathology theory (Cicchetti & Toth, 2005; Toth & Cicchetti, 2013).

I think that the support of theories to the research point of view needs to be completed and systematic arguments, not a one-sentence generalization.

Response:

Thank you for your valuable feedback. In response to your comment, we have added relevant literature references to support the claims made in Line 51, particularly regarding the importance of emotional support and a sense of security during childhood.

Additionally, we have expanded the theoretical argument by integrating developmental psychopathology theory (Cicchetti & Toth, 2005; Toth & Cicchetti, 2013). This theory emphasizes the role of both adaptive and maladaptive pathways established during childhood in shaping resilience and well-being trajectories across the lifespan. By incorporating this perspective along with developmental psychology and positive psychology, we have provided a more systematic and comprehensive theoretical foundation for understanding how positive childhood experiences (PCEs) influence subjective well-being (SWB) via resilience. We believe these revisions enhance the depth and coherence of the manuscript's theoretical support.

·Comment 4:

- Line 62 This is an analysis of why buffering stress is important for PCEs, but I have not found any psychological theory or literature to support it.

Response:

Thank you for your thoughtful comment. In response, we have now added relevant empirical studies to support the buffering effect of positive childhood experiences (PCEs). Specifically, the studies by Hall (2025) and Sousa et al. (2025) provide strong evidence that PCEs enhance emotional regulation and resilience, which in turn contribute to improved well-being. We believe these additions adequately support the buffering effect discussed in the manuscript.

·Comment 5:

- Line 67 As with the previous comment, I have not found any literature in support of this argument.

Response:

Thank you for your helpful comment. In response, we have added relevant literature to support the argument in Line 67 regarding the factors

·Comment 6:

- Line 69 This sentence contains a grammatical error.

Response:

Thank you for your comment. We have revised Line 69 of the original manuscript to correct the grammatical issue and improve clarity.

·Comment7:

- Still with the suggestion of theoretical support, I think it is not rigorous to support a research idea with just one sentence related to psychological theory.

Response:

Thank you for your comment. In response, we have expanded the theoretical support by integrating developmental psychopathology theory (Cicchetti & Toth, 2005; Toth & Cicchetti, 2013) along with developmental psychology and positive psychology. We have also added relevant literature and empirical evidence to support these theories, addressing the concern of relying on just one sentence. These revisions provide a more comprehensive and rigorous theoretical framework for our research.

·Comment 8:

- Line 86 It's incomprehensible that this sentence should appear here.

Response:

Thank you for your comment. In response, we have revised Line 86 of the original manuscript, which is now Line 102 after the revision. We have clarified and improved the clarity of the sentence by integrating additional theoretical and empirical support. The revised sentence now better highlights the impact of positive childhood experiences (PCEs) on psychological resilience and strengthens the overall argument.

·Comment 9:

- Line 88, this sentence seems redundant.

Response:

Thank you for your comment. Based on your suggestion, we have identified redundancy in Line 88 of the original manuscript. We have deleted this sentence in the revised version. Thank you for pointing this out.

·Comment 10:

- Line 96 recommends that manuscripts be carefully scrutinized for punctuation to meet publication requirements.

Is self-efficacy and emotional regulation the same concept as psychological resilience? I suspect that the cited literature does not support the research point of view.

Response:

Thank you for your comment. In response, we have carefully reviewed the manuscript for punctuation and made corrections to ensure compliance with publication requirements. Regarding your second point, we acknowledge that self-efficacy and emotional regulation are not the same as psychological resilience. While they are related constructs that may contribute to resilience, they represent distinct psychological mechanisms. To avoid conceptual overlap, we have revised the manuscript to clearly differentiate these concepts.

In addition, we have revised the cited literature to ensure that the references accurately support the research perspective. Specifically, we now focus on studies that highlight the role of psychological resilience in mediating the relationship between positive childhood experiences (PCEs) and subjective well-being (SWB). This revision strengthens the theoretical rigor of the manuscript and ensures that the cited evidence directly supports the argument.

·Comment11:

- Line 100 3 references to experimental studies, I only found the Turkish culture, the other one was not stated, so how did Line 100's concluding comments come about? As well I would like to know what is the significance of your repeated references to cross-cultural differences or stability to the ideas in your manuscript? Your study was conducted among Chinese subjects, how did cross-culturalization affect the Chinese subjects? Can the empirical studies cited here prove to be equally applicable in Chinese subjects?

Response:

Thank you for your comment. In response to your suggestion, we have removed the emphasis on cross-cultural validity and now focus more on the relevance of the mediation model in the context of Chinese subjects. This revision ensures that the argument is more relevant to our study population, without unnecessary cross-cultural comparisons. We believe this adjustment strengthens the clarity and applicability of the research.

Regarding your question about the applicability of the empirical studies cited in the context of Chinese subjects, we acknowledge that while the studies we cite primarily focus on populations from other cultural contexts, the core principles of psychological resilience and its mediating role in the PCEs-SWB link are likely to be applicable to Chinese subjects as well. However, we recognize the importance of cultural differences in shaping how PCEs and resilience are experienced and manifested, and as such, we emphasize that further research specific to the Chinese context is needed to fully confirm the applicability of these findings.

We believe that the concepts of psychological resilience and the relationship between PCEs and SWB are universal to some extent, but their expression and impact may vary based on cultural factors. Our study provides a valuable contribution to understanding how PCEs contribute to well-being in Chinese students, but further research is required to explore the cross-cultural applicability of these findings and how resilience plays out in other contexts.

·Comment 12:

- Line 102 I don't understand the point this paragraph is trying to make, why continue to make assumptions in a manuscript that have already been proven when it has already been written in the previous paragraph that the relationship between PCEs, psychological resilience, and SWB has already been proven?

Response:

Thank you for your comment. In response, we have revised the manuscript to remove redundancy and better reflect the focus of our study on the Chinese context. We have clarified that the relationship between PCEs, psychological resilience, and subjective well-being (SWB) has been established in previous research, and this study aims to explore how PCEs affect SWB directly and how psychological resilience mediates this relationship among Chinese university students. This revision strengthens the clarity of the study's objectives and emphasizes the unique contribution of our research.

·Comment 13:

- H1-H4 Please formulate your research hypothesis in more concise and scholarly language.

Response:

Thank you for your suggestion. In response, we have reformulated the research hypotheses (H1-H4) to present them in a more concise and scholarly manner. The revised hypotheses are now clearer and more aligned with academic writing standards.

·Comment 14:

- Line 124 Can't “obvious patterns (e.g., all the same answers)” in the manuscript represent an invalid questionnaire, and do all the same answers mean that the same options were chosen for all questions on the scale? Or are there consecutive q

Attachment Submitted filename: Response_to_Reviewers_auresp_2.docx

# Peer review: Decision Letter 2

**DOI:** 10.1371/journal.pone.0352258.r005

**Authors:** Shun Wang

23 Oct 2025

PONE-D-24-48467R2

Positive Childhood Experiences and Subjective Well-Being Among University Students in Yunnan Province, China: The Mediating Role of Psychological Resilience

PLOS ONE

Dear Dr. Yin,

Thank you for submitting your manuscript to PLOS ONE. After careful consideration, we feel that it has merit but does not fully meet PLOS ONE’s publication criteria as it currently stands. Therefore, we invite you to submit a revised version of the manuscript that addresses the points raised during the review process.

Please submit your revised manuscript by Dec 07 2025 11:59PM. If you will need more time than this to complete your revisions, please reply to this message or contact the journal office at plosone@plos.org. When you're ready to submit your revision, log on to https://www.editorialmanager.com/pone/ and select the 'Submissions Needing Revision' folder to locate your manuscript file.

Please include the following items when submitting your revised manuscript:

- A rebuttal letter that responds to each point raised by the academic editor and reviewer(s). You should upload this letter as a separate file labeled 'Response to Reviewers'.
- A marked-up copy of your manuscript that highlights changes made to the original version. You should upload this as a separate file labeled 'Revised Manuscript with Track Changes'.
- An unmarked version of your revised paper without tracked changes. You should upload this as a separate file labeled 'Manuscript'.

If you would like to make changes to your financial disclosure, please include your updated statement in your cover letter. Guidelines for resubmitting your figure files are available below the reviewer comments at the end of this letter.

If applicable, we recommend that you deposit your laboratory protocols in protocols.io to enhance the reproducibility of your results. Protocols.io assigns your protocol its own identifier (DOI) so that it can be cited independently in the future. For instructions see: https://journals.plos.org/plosone/s/submission-guidelines#loc-laboratory-protocols. Additionally, PLOS ONE offers an option for publishing peer-reviewed Lab Protocol articles, which describe protocols hosted on protocols.io. Read more information on sharing protocols at https://plos.org/protocols?utm_medium=editorial-email&utm_source=authorletters&utm_campaign=protocols.

We look forward to receiving your revised manuscript.

Kind regards,

Shun Wang

Academic Editor

PLOS ONE

Journal Requirements:

If the reviewer comments include a recommendation to cite specific previously published works, please review and evaluate these publications to determine whether they are relevant and should be cited. There is no requirement to cite these works unless the editor has indicated otherwise.

Additional Editor Comments:

While you have addressed many of the reviewers' comments, three critical issues remain in the revised draft:

1) Contribution Clarity: The manuscript repeatedly states that your findings are consistent with existing literature. Please clarify the novel theoretical or empirical contribution of your work.

2) SWB Measurement Justification: The decision to combine life satisfaction and affect into a single SWB measure requires strong theoretical or empirical support from the literature. If such support is lacking, we recommend treating them as two separate outcome variables in your analysis.

3) Variable Measurement Details: There is insufficient information on the measures for your key variables (Positive Childhood Experiences, Psychological Resilience, and SWB). Please list all survey questions for each measure, either in the main text or an appendix, to ensure clarity for a broad audience.

Please also address the minor issues listed in the separate section below.

1) Summary Statistics: Add a table (e.g., Table 1) presenting summary statistics (observations, mean, standard deviation, min, max) for all variables.

2) Clarify "invalid questionnaires": In line 139, explain what constitutes "other invalid questionnaires" in a footnote.

3) Formatting Consistency: Check and correct inconsistent font sizes (e.g., lines 105-106, 233-236) in the entire document.

4) Spacing Errors: Proofread the entire document to add missing spaces between words (e.g., line 78 before "Several factors").

[Note: HTML markup is below. Please do not edit.]

Reviewers' comments:

Reviewer's Responses to Questions

**Comments to the Author**

1. If the authors have adequately addressed your comments raised in a previous round of review and you feel that this manuscript is now acceptable for publication, you may indicate that here to bypass the “Comments to the Author” section, enter your conflict of interest statement in the “Confidential to Editor” section, and submit your "Accept" recommendation.

Reviewer #1: (No Response)

Reviewer #2: (No Response)

**********

2. Is the manuscript technically sound, and do the data support the conclusions?

The manuscript must describe a technically sound piece of scientific research with data that supports the conclusions. Experiments must have been conducted rigorously, with appropriate controls, replication, and sample sizes. The conclusions must be drawn appropriately based on the data presented.

Reviewer #1: (No Response)

Reviewer #2: Partly

**********

3. Has the statistical analysis been performed appropriately and rigorously?

Reviewer #1: Yes

Reviewer #2: N/A

**********

4. Have the authors made all data underlying the findings in their manuscript fully available?

The PLOS Data policy requires authors to make all data underlying the findings described in their manuscript fully available without restriction, with rare exception (please refer to the Data Availability Statement in the manuscript PDF file). The data should be provided as part of the manuscript or its supporting information, or deposited to a public repository. For example, in addition to summary statistics, the data points behind means, medians and variance measures should be available. If there are restrictions on publicly sharing data—e.g. participant privacy or use of data from a third party—those must be specified.

Reviewer #1: (No Response)

Reviewer #2: Yes

**********

5. Is the manuscript presented in an intelligible fashion and written in standard English?

PLOS ONE does not copyedit accepted manuscripts, so the language in submitted articles must be clear, correct, and unambiguous. Any typographical or grammatical errors should be corrected at revision, so please note any specific errors here.

Reviewer #1: Yes

Reviewer #2: Yes

**********

6. Review Comments to the Author

Please use the space provided to explain your answers to the questions above. You may also include additional comments for the author, including concerns about dual publication, research ethics, or publication ethics. (Please upload your review as an attachment if it exceeds 20,000 characters)

Reviewer #1: Comment 1

I appreciate that the authors have expanded the theoretical background and added several references clarifying the general associations between Positive Childhood Experiences (PCEs), resilience, and subjective well-being (SWB).

However, the sentence “By enhancing emotional regulation, problem-solving skills, and resilience, PCEs can act as a buffer against these stressors, ultimately promoting better mental health and well-being [18,19]” remains particularly important, as it appears to summarize the key theoretical assumption underlying the mediation model. As I understand it, this statement suggests that PCEs foster resilience, which in turn acts as a buffer against stressors and promotes better mental health and well-being — in other words, resilience is presented as the psychological mechanism through which PCEs exert their beneficial effects on well-being.

Given the centrality of this claim for the logic of the model, the supporting references should clearly substantiate it. Reference [19], which is a review, offers a useful overview of associations between PCEs, resilience, and well-being, but it does not explicitly explain how resilience functions as the buffering mechanism. Concerning reference [18], I admit that I was not able to retrieve it, but based on the title it does not seem directly relevant to this conceptual link. If this reference is indeed pertinent, I would kindly ask the authors to specify how it supports the proposed pathway.

I would therefore encourage the authors to verify the accuracy and relevance of reference [18], and to make explicit how the literature, particularly reference [19], supports this conceptual pathway. Specifically, the introduction should articulate more clearly how resilience operates as the mediating process through which PCEs exert their buffering effect on well-being.

Moreover, since the mediation effect of resilience was found to be statistically significant, it would be particularly important to strengthen the theoretical rationale underlying this pathway and to ensure that it is coherently reflected in the Discussion. In its current form, the Discussion states that PCEs may have boosted resilience via self-esteem and confidence; however, these constructs were not explicitly introduced a priori nor measured in the study. I would invite caution in introducing unmeasured constructs as explanatory mechanisms, unless they are clearly grounded in prior literature and framed as hypotheses for future research. Clarifying the theoretical sequence (how PCEs enhance resilience and how resilience, in turn, contributes to subjective well-being) would provide a more coherent interpretation of the findings and strengthen the overall conceptual contribution of the study.

Comment 2

I apologize for raising this point only at this stage, as it escaped my attention in the previous review round. Upon re-reading the manuscript, I realized that the operationalization of Subjective Well-Being (SWB) deserves further clarification. The current approach, in which SWB is computed as the sum of SWLS and PANAS total scores (with reverse-coded affect scores), may conflate conceptually distinct dimensions — cognitive (life satisfaction) and affective (positive and negative affect) components. Moreover, the two measures differ in item number, scale range, and variance, which can distort their relative contribution to the composite score. The moderate correlations typically observed between SWLS and PANAS further suggest that, although related, these constructs are not interchangeable; thus, a simple additive combination may alter the structure of the dependent variable and, in turn, influence the mediation results.

Additionally, it is not entirely clear what was included in the composite measure of SWB. The manuscript mentions “reverse-coded affect scores”, but does not specify whether both Positive and Negative Affect were used (with the latter reversed), or whether only reversed Negative Affect was combined with SWLS. These two approaches are conceptually different: excluding Positive Affect would not align with the standard tripartite model of SWB (Diener, 1984; Watson et al., 1988). I would therefore recommend that the authors clarify exactly which PANAS components were included and on what theoretical grounds.

I encourage the authors to provide a clear theoretical and empirical justification for this operationalization. In the absence of strong theoretical grounds, it might be advisable to (a) standardize the component scores before combining them, or (b) empirically test whether a single latent SWB factor can be supported, for instance through a confirmatory factor analysis verifying that SWLS, PA, and reversed NA load on a common factor with acceptable fit.

If this factor structure is not supported, the manuscript should acknowledge that the SWB components are distinct yet complementary, clarifying that the composite index reflects an overall summary of subjective well-being rather than a unidimensional latent construct. This clarification would strengthen the transparency and construct validity of the analyses without requiring major changes to the study design.

**********

7. PLOS authors have the option to publish the peer review history of their article (what does this mean?). If published, this will include your full peer review and any attached files.

If you choose “no”, your identity will remain anonymous but your review may still be made public.

**Do you want your identity to be public for this peer review?**   For information about this choice, including consent withdrawal, please see our Privacy Policy.

Reviewer #1: No

Reviewer #2: No

**********

[NOTE: If reviewer comments were submitted as an attachment file, they will be attached to this email and accessible via the submission site. Please log into your account, locate the manuscript record, and check for the action link "View Attachments". If this link does not appear, there are no attachment files.]

To ensure your figures meet our technical requirements, please review our figure guidelines: https://journals.plos.org/plosone/s/figures

You may also use PLOS’s free figure tool, NAAS, to help you prepare publication quality figures: https://journals.plos.org/plosone/s/figures#loc-tools-for-figure-preparation.

NAAS will assess whether your figures meet our technical requirements by comparing each figure against our figure specifications.

# Author comment: Author response to Decision Letter 3

**DOI:** 10.1371/journal.pone.0352258.r006

2 Nov 2025

Additional Editor Comments:

1) Contribution Clarity: The manuscript repeatedly states that your findings are consistent with existing literature. Please clarify the novel theoretical or empirical contribution of your work.

Response:

We sincerely appreciate this valuable comment. We agree that the previous version did not make the unique contribution of our study sufficiently explicit. In the revised manuscript, we have clarified both the empirical and methodological contributions in the Introduction and Discussion sections.

First, we now emphasize the empirical contribution of our study by situating it within the geographical context of China’s higher education system. As noted in prior research, most studies on subjective well-being among Chinese university students have been conducted in eastern and coastal regions, while empirical evidence from southwestern provinces remains scarce. By analyzing data from four universities in Yunnan Province, our study helps to broaden the geographical coverage of well-being research in China and provides regionally grounded evidence on the PCE–resilience–SWB pathway. This addition enhances the external validity and contextual representativeness of our findings.

Second, in our mediation analysis, we included key socio-economic covariates—place of origin (urban/rural), year of study, and family income—to test whether the PCE–resilience–SWB mechanism would remain significant after controlling for demographic influences. The results demonstrated that this mediation pattern persisted when these socio-economic factors were controlled, indicating the robustness of the mechanism and improving the analytical rigor of our model.

2) SWB Measurement Justification: The decision to combine life satisfaction and affect into a single SWB measure requires strong theoretical or empirical support from the literature. If such support is lacking, we recommend treating them as two separate outcome variables in your analysis.

Response:

We sincerely appreciate the editor’s careful attention to the operationalization of Subjective Well-Being (SWB). In response, we have substantially revised the Methods section to provide a clearer theoretical and empirical justification for computing a composite SWB score.

Our operationalization is grounded in the tripartite model of SWB proposed by Diener (1984)[1] and further elaborated by Diener et al. (1999)[2], which conceptualizes SWB as comprising cognitive evaluations (life satisfaction) and affective components (positive and negative affect). This integrated perspective has become a widely accepted framework in the study of well-being.

Following this model and prior empirical research[2-4], we computed a composite SWB score consistent with the empirical method used by Weinstein and Ryan (2010)[5]. Specifically, scores for each scale were standardized, and the composite SWB index was calculated using the formula:

SWB = general PA – general NA + life satisfaction.

This standardized computation balances the contributions of affective and cognitive domains and has been shown to validly represent trait-level well-being across diverse samples (Weinstein & Ryan, 2010)[5]. We have clarified this method in Section 2.2.3 (Methods) of the revised manuscript and emphasized that the composite SWB score serves as a summary index integrating both affective balance (PA–NA) and cognitive evaluation (life satisfaction)

3)Variable Measurement Details: There is insufficient information on the measures for your key variables (Positive Childhood Experiences, Psychological Resilience, and SWB). Please list all survey questions for each measure, either in the main text or an appendix, to ensure clarity for a broad audience.

Response:

We appreciate the editor’s thoughtful suggestion. To enhance measurement transparency and reproducibility, we have now included the full item sets of all three key instruments—Positive Childhood Experiences (BCEs), Psychological Resilience (CD-RISC), and Subjective Well-Being (SWLS + PANAS)—in a newly added Appendix A.

The Methods section (2.2.1–2.2.3) now also provides concise descriptions of each scale, including response format, scoring procedures, reliability coefficients, and relevant references.

Minor Comment :

1) Summary Statistics: Add a table (e.g., Table 1) presenting summary statistics (observations, mean, standard deviation, min, max) for all variables.

Response：

We sincerely thank the editor for this helpful suggestion. In the revised manuscript, we have expanded Table 1 to include the minimum and maximum values for all main variables (Positive Childhood Experiences, Psychological Resilience, and Subjective Well-Being), in addition to the existing means and standard deviations.

2) Clarify "invalid questionnaires": In line 139, explain what constitutes "other invalid questionnaires" in a footnote.

Response：

We appreciate the editor’s careful attention to methodological clarity. To make our data screening criteria more explicit, we have added a footnote in Section 2.1 to specify what was meant by “other invalid questionnaires.”

3) Formatting Consistency: Check and correct inconsistent font sizes (e.g., lines 105-106, 233-236) in the entire document.

Response：

We are grateful to the editor for noticing these formatting inconsistencies.We have carefully reviewed and corrected all formatting inconsistencies throughout the manuscript, including font size and style.

4) Spacing Errors: Proofread the entire document to add missing spaces between words (e.g., line 78 before "Several factors").

Response：

We thank the editor for this observation. We have thoroughly proofread the entire manuscript and corrected all spacing issues, including missing spaces between words and after punctuation marks.

Reviewer #1:

Comment 1

The reviewer correctly highlights that the sentence “By enhancing emotional regulation, problem-solving skills, and resilience, PCEs can act as a buffer against these stressors, ultimately promoting better mental health and well-being [18,19]” appears central to the proposed mediation model. However, the cited references (particularly reference [18]) lack direct support for this mediation pathway. The reviewer requests a clearer theoretical articulation of how Psychological Resilience functions as the mechanism linking PCEs and SWB, both in the Introduction and Discussion.

Response:

We are very grateful to the reviewer for this insightful comment regarding the theoretical foundation of the mediation pathway linking Positive Childhood Experiences (PCEs), resilience, and Subjective Well-Being (SWB).

In response, we have carefully revised both the Introduction and Discussion sections to articulate more clearly how resilience functions as the psychological mechanism through which PCEs exert their positive effects on well-being.

Specifically, we have removed the ambiguous citation of Hall (2025) [former reference 18], as it did not offer sufficient empirical or theoretical grounding for the mediation pathway. In its place, we added and emphasized relevant literature that directly supports the mediating role of resilience, including:

“Masten (2001) and Luthar et al. (2000), who explain resilience as an adaptive process supported by early positive experiences;”

“Narayan et al. (2018) and Sousa et al. (2025), who show that PCEs promote psychological resilience across development;”

“Kocatürk & Çiçek (2023) and Melih & Oktay (2024), who provide empirical support for the mediating role of resilience between PCEs and well-being among university students.”

We have also revised the key sentence in the Introduction to more clearly reflect this developmental mechanism.

Furthermore, in the Discussion, we have avoided introducing unmeasured constructs (such as self-esteem or confidence) and instead focused on resilience as the empirically supported mediator.

These revisions strengthen the conceptual clarity and theoretical coherence of the mediation model, and we sincerely appreciate the reviewer’s valuable input, which has helped us refine the manuscript substantially.

Comment2

“It is not entirely clear what was included in the composite measure of SWB... The manuscript mentions ‘reverse-coded affect scores,’ but does not specify whether both Positive and Negative Affect were used (with the latter reversed), or whether only reversed Negative Affect was combined with SWLS…”

Response:

We appreciate the reviewer’s close reading and constructive suggestion. We have now revised the text to explicitly state that the composite SWB score was computed from three standardized components: Positive Affect (PA), Negative Affect (NA) (both assessed via PANAS), and Life Satisfaction (SWLS). The affective component was operationalized as PA minus NA, following the tripartite model of SWB (Diener, 1984; Watson et al., 1988)[1, 6], which conceptualizes cognitive and affective dimensions as distinct yet interrelated aspects of well-being.

To ensure consistency across instruments with different item numbers and scale ranges, each scale score was standardized before aggregation. The final SWB index was computed using the following formula: SWB = general PA – general NA + life satisfaction.

This computation follows the empirical approach of Weinstein and Ryan (2010)[5], who applied the same formula after standardizing individual scales, demonstrating its empirical validity and conceptual coherence. Importantly, we have clarified that the composite SWB serves as a theoretically informed composite index—an integrative measure of cognitive and affective well-being. This clarification has been incorporated in the revised Methods section (2.2.3).

1. Diener E. Subjective well -being. Psychological Bulletin. 1984;95(3):542-75. doi: 10.1037/0033-2909.95.3.542. PubMed PMID: WOS:A1984SR64000009.

2. Diener E, Suh EM, Lucas RE, Smith HL. Subjective well-being: Three decades of progress. Psychological Bulletin. 1999;125(2):276-302. doi: 10.1037//0033-2909.125.2.276. PubMed PMID: WOS:000078980900007.

3. Argyle M, Crossland J. The dimensions of positive emotions. British Journal of Social Psychology. 1987;26(2):127-37. doi: 10.1111/j.2044-8309.1987.tb00773.x.

4. Diener E, Emmons RA. The independence of positive and negative affect. Journal of personality and social psychology. 1984;47(5):1105. doi: 10.1037/0022-3514.47.5.1105.

5. Weinstein N, Ryan RM. When helping helps: autonomous motivation for prosocial behavior and its influence on well-being for the helper and recipient. Journal of personality and social psychology. 2010;98(2):222. doi: 10.1037/a0016984.

6. Watson D, Clark LA, Tellegen A. Development and validation of brief measures of positive and negative affect: the PANAS scales. Journal of personality and social psychology. 1988;54(6):1063. doi: 10.1037/0022-3514.54.6.1063.

Attachment Submitted filename: renamed_e6b61.docx

# Peer review: Decision Letter 3

**DOI:** 10.1371/journal.pone.0352258.r007

**Authors:** Denise Evans

11 Mar 2026

PONE-D-24-48467R3

Positive Childhood Experiences and Subjective Well-Being Among University Students in Yunnan Province, China: The Mediating Role of Psychological Resilience

PLOS One

Dear Dr. Yin,

Thank you for submitting your manuscript to PLOS ONE. After careful consideration, we feel that it has merit but does not fully meet PLOS ONE’s publication criteria as it currently stands. Therefore, we invite you to submit a revised version of the manuscript that addresses the points raised during the review process.

Please submit your revised manuscript by Apr 25 2026 11:59PM. If you will need more time than this to complete your revisions, please reply to this message or contact the journal office at plosone@plos.org. When you're ready to submit your revision, log on to https://www.editorialmanager.com/pone/ and select the 'Submissions Needing Revision' folder to locate your manuscript file.

Please include the following items when submitting your revised manuscript:

- A letter that responds to each point raised by the academic editor and reviewer(s). You should upload this letter as a separate file labeled 'Response to Reviewers'.
- A marked-up copy of your manuscript that highlights changes made to the original version. You should upload this as a separate file labeled 'Revised Manuscript with Track Changes'.
- An unmarked version of your revised paper without tracked changes. You should upload this as a separate file labeled 'Manuscript'.

If you would like to make changes to your financial disclosure, please include your updated statement in your cover letter. Guidelines for resubmitting your figure files are available below the reviewer comments at the end of this letter.

If applicable, we recommend that you deposit your laboratory protocols in protocols.io to enhance the reproducibility of your results. Protocols.io assigns your protocol its own identifier (DOI) so that it can be cited independently in the future. For instructions see: https://journals.plos.org/plosone/s/submission-guidelines#loc-laboratory-protocols. Additionally, PLOS ONE offers an option for publishing peer-reviewed Lab Protocol articles, which describe protocols hosted on protocols.io. Read more information on sharing protocols at https://plos.org/protocols?utm_medium=editorial-email&utm_source=authorletters&utm_campaign=protocols.

We look forward to receiving your revised manuscript.

Kind regards,

Denise Evans, PhD

Academic Editor

PLOS One

Journal Requirements:

If the reviewer comments include a recommendation to cite specific previously published works, please review and evaluate these publications to determine whether they are relevant and should be cited. There is no requirement to cite these works unless the editor has indicated otherwise.

[Note: HTML markup is below. Please do not edit.]

Reviewers' comments:

Reviewer's Responses to Questions

**Comments to the Author**

1. If the authors have adequately addressed your comments raised in a previous round of review and you feel that this manuscript is now acceptable for publication, you may indicate that here to bypass the “Comments to the Author” section, enter your conflict of interest statement in the “Confidential to Editor” section, and submit your "Accept" recommendation.

Reviewer #1: All comments have been addressed

Reviewer #3: (No Response)

**********

2. Is the manuscript technically sound, and do the data support the conclusions?

The manuscript must describe a technically sound piece of scientific research with data that supports the conclusions. Experiments must have been conducted rigorously, with appropriate controls, replication, and sample sizes. The conclusions must be drawn appropriately based on the data presented.

Reviewer #1: Yes

Reviewer #3: Yes

**********

3. Has the statistical analysis been performed appropriately and rigorously?

Reviewer #1: Yes

Reviewer #3: Yes

**********

4. Have the authors made all data underlying the findings in their manuscript fully available?

The PLOS Data policy requires authors to make all data underlying the findings described in their manuscript fully available without restriction, with rare exception (please refer to the Data Availability Statement in the manuscript PDF file). The data should be provided as part of the manuscript or its supporting information, or deposited to a public repository. For example, in addition to summary statistics, the data points behind means, medians and variance measures should be available. If there are restrictions on publicly sharing data—e.g. participant privacy or use of data from a third party—those must be specified.

Reviewer #1: (No Response)

Reviewer #3: Yes

**********

5. Is the manuscript presented in an intelligible fashion and written in standard English?

PLOS ONE does not copyedit accepted manuscripts, so the language in submitted articles must be clear, correct, and unambiguous. Any typographical or grammatical errors should be corrected at revision, so please note any specific errors here.

Reviewer #1: (No Response)

Reviewer #3: Yes

**********

6. Review Comments to the Author

Please use the space provided to explain your answers to the questions above. You may also include additional comments for the author, including concerns about dual publication, research ethics, or publication ethics. (Please upload your review as an attachment if it exceeds 20,000 characters)

Reviewer #1: The authors have addessed all the comments and issues I've raised. I think that the paper is now suitable for publication

Reviewer #3: Discussion section refers to other studies but in some cases, these other studies are not explained in relation to this study findings. There are still grammatical errors and spacing issues within the document. The lack of ethics clearance is a concern as there are some items in the questionnaires that are invasive.

**********

7. PLOS authors have the option to publish the peer review history of their article (what does this mean?). If published, this will include your full peer review and any attached files.

If you choose “no”, your identity will remain anonymous but your review may still be made public.

**Do you want your identity to be public for this peer review?**   For information about this choice, including consent withdrawal, please see our Privacy Policy.

Reviewer #1: No

Reviewer #3: No

**********

[NOTE: If reviewer comments were submitted as an attachment file, they will be attached to this email and accessible via the submission site. Please log into your account, locate the manuscript record, and check for the action link "View Attachments". If this link does not appear, there are no attachment files.]

To ensure your figures meet our technical requirements, please review our figure guidelines: https://journals.plos.org/plosone/s/figures

You may also use PLOS’s free figure tool, NAAS, to help you prepare publication quality figures: https://journals.plos.org/plosone/s/figures#loc-tools-for-figure-preparation.

NAAS will assess whether your figures meet our technical requirements by comparing each figure against our figure specifications.

# Author comment: Author response to Decision Letter 4

**DOI:** 10.1371/journal.pone.0352258.r008

18 Mar 2026

Response to Reviewers

We sincerely thank the Academic Editor and the reviewers for their valuable comments and constructive suggestions. We have carefully revised the manuscript in accordance with all comments. All revisions have been clearly marked in the tracked-changes version, and the specific modifications are detailed below.

We would like to sincerely thank Reviewer #1 for the positive evaluation of our manuscript and for confirming that all previous comments have been adequately addressed. We greatly appreciate the reviewer’s time and constructive feedback throughout the review process.

Reviewer #3:

Comment 1:

Discussion section refers to other studies but in some cases these other studies are not explained in relation to this study findings.

Response:

We sincerely thank the reviewer for this insightful and helpful comment, which has significantly improved the clarity and depth of the Discussion section. In response, we have carefully revised the Discussion to more clearly connect the cited literature with the findings of the present study.

Specifically, we reorganized each subsection of the Discussion to first restate the key findings of this study and then interpret these findings in relation to existing literature. We also strengthened the explanation of the underlying mechanisms linking Positive Childhood Experiences (PCEs), psychological resilience, and Subjective Well-Being (SWB).

In particular, we have substantially revised the section on the mediating role of psychological resilience to clarify how resilience functions as a pathway linking PCEs and SWB. We now explicitly explain the partial mediation effect observed in this study and discuss possible additional mechanisms that may account for the remaining direct effect.

These revisions have enhanced the theoretical interpretation of our findings and strengthened the overall contribution of the study. The revised content can be found in Section 4 (Discussion), particularly in subsections 4.1–4.4.

Comment 2:

There are still grammatical errors and spacing issues within the document.

Response:

We sincerely thank the reviewer for pointing this out. We have carefully proofread the entire manuscript and corrected grammatical errors and spacing issues throughout the text. In particular, we have revised punctuation, spacing between words and references, and minor typographical errors to improve clarity and readability.

Comment 3:

The lack of ethics clearance is a concern as there are some items in the questionnaires that are invasive.

Response:

We sincerely thank the reviewer for raising this important concern, which has helped us improve the clarity and transparency of the ethical statement.

In response, we have added a detailed ethical statement in the Methods section (Section 2.1 Participants and Procedures). The revised manuscript now clearly states that the study protocol was reviewed by the Ethics Committee of Yunnan Normal University and was granted exemption from full ethical review (Approval No. 2023012), as the study involved an anonymous questionnaire survey of adult participants and posed minimal risk.

We have also clarified that no personally identifiable information was collected. In addition, we explicitly state that participation was voluntary, informed consent was obtained prior to participation, and participants had the right to withdraw from the study at any time without penalty.

These revisions ensure that the ethical considerations of the study are clearly presented and fully comply with international publication standards.

We sincerely appreciate the editor’s and reviewers’ time and valuable feedback. We believe that the revisions have addressed all concerns and significantly improved the manuscript, and we hope that the revised version is now suitable for publication in PLOS ONE.

Attachment Submitted filename: Response_to_Reviewers_auresp_4.docx

# Editor report: Decision Letter 4

**DOI:** 10.1371/journal.pone.0352258.r009

**Authors:** Denise Evans

15 Apr 2026

PONE-D-24-48467R4

Positive Childhood Experiences and Subjective Well-Being Among University Students in Yunnan Province, China: The Mediating Role of Psychological Resilience

PLOS One

Dear Dr. Yin,

Thank you for submitting your manuscript to PLOS ONE. After careful consideration, we feel that it has merit but does not fully meet PLOS ONE’s publication criteria as it currently stands. Therefore, we invite you to submit a revised version of the manuscript that addresses the points raised during the review process.

Please submit your revised manuscript by May 30 2026 11:59PM. If you will need more time than this to complete your revisions, please reply to this message or contact the journal office at plosone@plos.org. When you're ready to submit your revision, log on to https://www.editorialmanager.com/pone/ and select the 'Submissions Needing Revision' folder to locate your manuscript file.

Please include the following items when submitting your revised manuscript:

- A letter that responds to each point raised by the academic editor and reviewer(s). You should upload this letter as a separate file labeled 'Response to Reviewers'.
- A marked-up copy of your manuscript that highlights changes made to the original version. You should upload this as a separate file labeled 'Revised Manuscript with Track Changes'.
- An unmarked version of your revised paper without tracked changes. You should upload this as a separate file labeled 'Manuscript'.

If you would like to make changes to your financial disclosure, please include your updated statement in your cover letter. Guidelines for resubmitting your figure files are available below the reviewer comments at the end of this letter.

If applicable, we recommend that you deposit your laboratory protocols in protocols.io to enhance the reproducibility of your results. Protocols.io assigns your protocol its own identifier (DOI) so that it can be cited independently in the future. For instructions see: https://journals.plos.org/plosone/s/submission-guidelines#loc-laboratory-protocols. Additionally, PLOS ONE offers an option for publishing peer-reviewed Lab Protocol articles, which describe protocols hosted on protocols.io. Read more information on sharing protocols at https://plos.org/protocols?utm_medium=editorial-email&utm_source=authorletters&utm_campaign=protocols.

As the corresponding author, your ORCID iD is verified in the submission system and will appear in the published article. PLOS supports the use of ORCID, and we encourage all coauthors to register for an ORCID iD and use it as well. Please encourage your coauthors to verify their ORCID iD within the submission system before final acceptance, as unverified ORCID iDs will not appear in the published article. *Only*   the individual author can complete the verification step; PLOS staff *cannot*   verify ORCID iDs on behalf of authors.

We look forward to receiving your revised manuscript.

Kind regards,

Denise Evans, PhD

Academic Editor

PLOS One

Journal Requirements:

If the reviewer comments include a recommendation to cite specific previously published works, please review and evaluate these publications to determine whether they are relevant and should be cited. There is no requirement to cite these works unless the editor has indicated otherwise.

Additional Editor Comments:

Please confirm whether the study has received ethics clearance.

On page 7 of the manuscript, it states that ‘The study protocol was reviewed by the ethics committee of Yunnan Normal University.’ However, it remains unclear why the study qualified for exemption, given that it involved direct interaction with participants and the collection of informed consent, even with the use of anonymous questionnaires. Kindly upload the official communication from the ethics committee indicating that the protocol was reviewed and exempted and the reasons for exemption.

[Note: HTML markup is below. Please do not edit.]

[NOTE: If reviewer comments were submitted as an attachment file, they will be attached to this email and accessible via the submission site. Please log into your account, locate the manuscript record, and check for the action link "View Attachments". If this link does not appear, there are no attachment files.]

To ensure your figures meet our technical requirements, please review our figure guidelines: https://journals.plos.org/plosone/s/figures

You may also use PLOS’s free figure tool, NAAS, to help you prepare publication quality figures: https://journals.plos.org/plosone/s/figures#loc-tools-for-figure-preparation.

NAAS will assess whether your figures meet our technical requirements by comparing each figure against our figure specifications.

# Author comment: Author response to Decision Letter 5

**DOI:** 10.1371/journal.pone.0352258.r010

20 Apr 2026

We sincerely thank you for your careful evaluation of our manuscript and for your constructive comments and suggestions. We have carefully revised the manuscript accordingly. Below we provide our point-by-point response to the editorial comment.

Response to the Academic Editor

Comment :

Please confirm whether the study has received ethics clearance.

On page 7 of the manuscript, it states that ‘The study protocol was reviewed by the ethics committee of Yunnan Normal University.’ However, it remains unclear why the study qualified for exemption, given that it involved direct interaction with participants and the collection of informed consent, even with the use of anonymous questionnaires. Kindly upload the official communication from the ethics committee indicating that the protocol was reviewed and exempted and the reasons for exemption.

Response:

Thank you very much for this important comment. We agree that the ethics statement in the previous version of the manuscript was not sufficiently detailed.

We would like to clarify that this study was reviewed by the Ethics Review Group of the Applied Psychology Program, Faculty of Education, Yunnan Normal University, and was granted an ethics exemption on August 5, 2023 (No. 2023012).

The exemption was granted because the study involved a fully anonymous, non-invasive questionnaire survey. No directly or indirectly identifiable personal information was collected, no sensitive personal information was involved, and the study included only general adult university students rather than vulnerable populations. In addition, the study involved no physical, clinical, psychological, or medical intervention, and the overall level of risk was considered no greater than minimal. Participants read the informed consent statement before voluntarily completing and submitting the anonymous questionnaire, which constituted informed consent under this study design.

After receiving the editorial comment, we communicated with the university’s Ethics Review Group and obtained a formal certificate based on the original ethics review record. The certificate dated April 17, 2026 is an official documentary confirmation of the original review decision made on August 5, 2023.

To support this clarification, we have uploaded the following four attachments:

Original Ethical Review Application (English)

Original Ethical Review Application (Chinese)

Certificate of Ethics Review Exemption (Chinese)

Certificate of Ethics Review Exemption (English)

We have also revised the ethics statement in the manuscript accordingly to make the review status and the basis for exemption clearer.

Revised text in the manuscript:

“The study protocol was reviewed by the Ethics Review Group of the Applied Psychology Program, Faculty of Education, Yunnan Normal University, and was granted an ethics exemption on August 5, 2023 (No. 2023012). The study involved a fully anonymous, non-invasive questionnaire survey of adult university students, collected no directly or indirectly identifiable personal information or sensitive personal information, and posed no greater than minimal risk to participants. Participants read the informed consent statement before voluntarily completing and submitting the anonymous questionnaire, which constituted informed consent under this study design. Participants were informed that they could withdraw at any time without providing any reason.”

Location in revised manuscript:

Methods section, page 7, lines 162—170.

We thank the Academic Editor again for this helpful comment, which has enabled us to improve the clarity and transparency of the manuscript. We hope that the revised version is now suitable for publication in PLOS ONE.

Attachment Submitted filename: Response_to_Reviewers_auresp_5.docx

# Peer review: Decision Letter 5

**DOI:** 10.1371/journal.pone.0352258.r011

**Authors:** Petri Böckerman, Ching Sin Siau, Shun Wang, Denise Evans, Denise Evans, Denise Evans

8 Jun 2026

Positive Childhood Experiences and Subjective Well-Being Among University Students in Yunnan Province, China: The Mediating Role of Psychological Resilience

PONE-D-24-48467R5

Dear Dr. Yin,

We’re pleased to inform you that your manuscript has been judged scientifically suitable for publication and will be formally accepted for publication once it meets all outstanding technical requirements.

Within one week, you’ll receive an e-mail detailing the required amendments. When these have been addressed, you’ll receive a formal acceptance letter and your manuscript will be scheduled for publication.

An invoice will be generated when your article is formally accepted. Please note, if your institution has a publishing partnership with PLOS and your article meets the relevant criteria, all or part of your publication costs will be covered. Please make sure your user information is up-to-date by logging into Editorial Manager at Editorial Manager® and clicking the ‘Update My Information' link at the top of the page. For questions related to billing, please contact billing support.

If your institution or institutions have a press office, please notify them about your upcoming paper to help maximize its impact. If they’ll be preparing press materials, please inform our press team as soon as possible -- no later than 48 hours after receiving the formal acceptance. Your manuscript will remain under strict press embargo until 2 pm Eastern Time on the date of publication. For more information, please contact onepress@plos.org.

Kind regards,

Denise Evans, PhD

Academic Editor

PLOS One

Additional Editor Comments (optional):

Reviewers' comments:

Reviewer's Responses to Questions

**Comments to the Author**

1. If the authors have adequately addressed your comments raised in a previous round of review and you feel that this manuscript is now acceptable for publication, you may indicate that here to bypass the “Comments to the Author” section, enter your conflict of interest statement in the “Confidential to Editor” section, and submit your "Accept" recommendation.

Reviewer #3: All comments have been addressed

**********

2. Is the manuscript technically sound, and do the data support the conclusions?

The manuscript must describe a technically sound piece of scientific research with data that supports the conclusions. Experiments must have been conducted rigorously, with appropriate controls, replication, and sample sizes. The conclusions must be drawn appropriately based on the data presented.

Reviewer #3: Yes

**********

3. Has the statistical analysis been performed appropriately and rigorously?

Reviewer #3: Yes

**********

4. Have the authors made all data underlying the findings in their manuscript fully available?

The PLOS Data policy requires authors to make all data underlying the findings described in their manuscript fully available without restriction, with rare exception (please refer to the Data Availability Statement in the manuscript PDF file). The data should be provided as part of the manuscript or its supporting information, or deposited to a public repository. For example, in addition to summary statistics, the data points behind means, medians and variance measures should be available. If there are restrictions on publicly sharing data—e.g. participant privacy or use of data from a third party—those must be specified.

Reviewer #3: Yes

**********

5. Is the manuscript presented in an intelligible fashion and written in standard English?

PLOS ONE does not copyedit accepted manuscripts, so the language in submitted articles must be clear, correct, and unambiguous. Any typographical or grammatical errors should be corrected at revision, so please note any specific errors here.

Reviewer #3: Yes

**********

6. Review Comments to the Author

Please use the space provided to explain your answers to the questions above. You may also include additional comments for the author, including concerns about dual publication, research ethics, or publication ethics. (Please upload your review as an attachment if it exceeds 20,000 characters)

Reviewer #3: This article has been been peer reviewed by me before and this was the last issue to be addressed. The ethics issues have since been addressed.

**********

7. PLOS authors have the option to publish the peer review history of their article (what does this mean?). If published, this will include your full peer review and any attached files.

If you choose “no”, your identity will remain anonymous but your review may still be made public.

**Do you want your identity to be public for this peer review?**   For information about this choice, including consent withdrawal, please see our Privacy Policy.

Reviewer #3: No

**********

# Editor report: Acceptance letter

**DOI:** 10.1371/journal.pone.0352258.r012

**Authors:** Petri Böckerman, Ching Sin Siau, Shun Wang, Denise Evans, Denise Evans, Denise Evans

PONE-D-24-48467R5

PLOS One

Dear Dr. Yin,

I'm pleased to inform you that your manuscript has been deemed suitable for publication in PLOS One. Congratulations! Your manuscript is now being handed over to our production team.

At this stage, our production department will prepare your paper for publication. This includes ensuring the following:

* All references, tables, and figures are properly cited

* All relevant supporting information is included in the manuscript submission,

* There are no issues that prevent the paper from being properly typeset

You will receive further instructions from the production team, including instructions on how to review your proof when it is ready. Please keep in mind that we are working through a large volume of accepted articles, so please give us a few days to review your paper and let you know the next and final steps.

Lastly, if your institution or institutions have a press office, please let them know about your upcoming paper now to help maximize its impact. If they'll be preparing press materials, please inform our press team within the next 48 hours. Your manuscript will remain under strict press embargo until 2 pm Eastern Time on the date of publication. For more information, please contact onepress@plos.org.

You will receive an invoice from PLOS for your publication fee after your manuscript has reached the completed accept phase. If you receive an email requesting payment before acceptance or for any other service, this may be a phishing scheme. Learn how to identify phishing emails and protect your accounts at https://explore.plos.org/phishing.

If we can help with anything else, please email us at customercare@plos.org.

Thank you for submitting your work to PLOS ONE and supporting open access.

Kind regards,

PLOS ONE Editorial Office Staff

on behalf of

Dr. Denise Evans

Academic Editor

PLOS One
